# The Odorant Binding Protein 6 Expressed in Sensilla Chaetica Displays Preferential Binding Affinity to Host Plants Volatiles in *Ectropis obliqua*

**DOI:** 10.3389/fphys.2018.00534

**Published:** 2018-05-17

**Authors:** Long Ma, Zhaoqun Li, Wanna Zhang, Xiaoming Cai, Zongxiu Luo, Yongjun Zhang, Zongmao Chen

**Affiliations:** ^1^Jiangxi Key Laboratory of Bioprocess Engineering, College of Life Sciences, Jiangxi Science & Technology Normal University, Nanchang, China; ^2^Tea Research Institute, Chinese Academy of Agricultural Sciences, Hangzhou, China; ^3^Institute of Entomology, Jiangxi Agricultural University, Nanchang, China; ^4^State Key Laboratory for Biology of Plant Diseases and Insect Pests, Institute of Plant Protection, Chinese Academy of Agricultural Sciences, Beijing, China

**Keywords:** *Ectropis obliqua*, odorant-binding protein, immunolocalization, sensilla chaetica, fluorescence competition assay

## Abstract

The monophagous tea geometrid *Ectropis obliqua* selectively feed on tea plants, requiring the specialized chemosensory system to forage for certain host. A deep insight into the molecular basis would accelerate the design of insect-behavior-modifying stimuli. In the present study, we focused on the odorant-binding protein 6 (EoblOBP6) with the high abundance in legs transcriptome of *E. obliqua* moths. qRT-PCR coupled with western blot analyses revealed the dual expression pattern of EoblOBP6 in antennae and legs. Cellular immunolocalization indicated that EoblOBP6 was predominantly labeled in the outer sensillum lymph of uniporous sensilla chaetica, which is not innervated by sensory neurons. No specific staining was observed in other sensillum types. The fluorescence competition assay showed a relatively narrow binding spectrum of recombinant EoblOBP6. EoblOBP6 could not only bind with intact tea plant volatiles benzaldehyde but also display high binding ability to nerolidol and α-farnesene which are tea plant volatiles dramatically induced by herbivore infestation. Besides, EoblOBP6 tightly bound to the aversive bitter alkaloid berberine. Taken together, EoblOBP6 displayed an unusual expression in sensilla chaetica, exhibited the potential involvement in olfaction and gustation, and may play a functional role in host location of female *E. obliqua* moths.

## Introduction

Moths have evolved a sophisticated olfactory system to detect various semiochemicals, guiding their feeding, mating, predator avoidance and oviposition behaviors. The hydrophobic odorant and taste molecules diffuse through pores in the sensillum surface and enter the sensillum lymph (Steinbrecht et al., 1995), after which they are delivered by carrier proteins to receptors located within the dendritic membrane of sensory neurons (Pelosi, 1996). During this process, the high sensitive and selective insect olfaction depend heavily on two types of proteins, the carrier proteins and the olfactory receptors (ORs) (Große-Wilde et al., 2006; Benton et al., 2007; Forstner et al., 2009). Insect odorant binding proteins (OBPs) are small soluble carrier proteins (∼15 kDa) and are characterized by a specific domain that constitutes six α-helices joined by two-four disulphide bridges (Leal et al., 1999; Tegoni et al., 2004; Pelosi et al., 2014). Studies by *in situ* hybridization and immunolocalization have confirmed that OBPs are synthesized by the auxiliary cells surrounding neurons and are subsequently secreted into the sensillum lymph in a high concentration (Michael, 2000; de Santis et al., 2006). Involved in the initial steps of odorant reception, insect OBPs are presumed to bind, solubilize and transport the hydrophobic odorants through an aqueous lymph, and eventually reach sensory dendrites, where they activate the membrane-bound ORs (Pelosi et al., 2006).

Since the first identification of insect OBPs in the silkmoth *Antheraea polyphemus* where they bind with sex pheromones (Vogt and Riddiford, 1981), numerous OBPs have been investigated for their indispensable roles and potential involvement in olfaction (Berg and Ziegelberger, 1991; Xu et al., 2005; Biessmann et al., 2010; Wang et al., 2013). In *Acyrthosiphon pisum*, the repellent behavior to the alarm pheromone (*E*)-β-farnesene (EBF) was significantly impaired after dual knockdown of *ApisOBP3* and *ApisOBP7* which were known to bind EBF (Sun Y.F. et al., 2012; Zhang et al., 2017). Similarly, in *Helicoverpa armigera* and *Chilo suppressalis*, the CRISPR/Cas9 mediated pheromone binding proteins (PBPs) mutagenesis resulted in the severely impaired responses to sex pheromone components in male adults (Dong et al., 2017; Ye et al., 2017). Moreover, the behavioral assays in aphids (Qiao et al., 2009; Sun Y.F. et al., 2012) and *Drosophila* mutants (Matsuo et al., 2007; Swarup et al., 2011) also revealed that OBPs are truly engaged in the semiochemical perception. However, till now, the mode of action of these proteins remains incomplete. The *Drosophila* mutants lacking LUSH (OBP76a) were insensitive to their sex pheromone 11-*cis*-vaccenyl acetate (cVA), proving an indispensable role of LUSH in pheromone signal transduction (Xu et al., 2005). Likewise, LUSH is proved to be required for response to VA when VA receptors are expressed in non-T1 neurons (Ha and Smith, 2006). Laughlin et al. (2008) further concluded that LUSH bound to cVA forms an OBP-odorant complex that activates the pheromone-sensitive neuron. But later research showed that high concentration of pheromone can *per se* induce neuronal activity when devoid of LUSH, indicating that pheromone molecules alone directly activate its neuronal receptors (Gomez-Diaz et al., 2013). Besides, studies involving the combinations of PBP and pheromone receptors (PRs) from *Chilo suppressalis* and *Bombyx mori* indicate that PRs sensitivity to pheromones is greatly enhanced when co-expressed with PBPs (Syed et al., 2010; Chang et al., 2015). A reasonable explanation is that the presence of OBPs can increase the sensitivity of olfactory receptors to odorants (Große-Wilde et al., 2006; Sun et al., 2013; Chang et al., 2015).

Most insect OBPs are exclusively or dominantly expressed in antennae, and many studies have documented the different arrangement of OBPs in certain types of antennal sensilla (Steinbrecht et al., 1995; Shanbhag et al., 2001; Gu et al., 2013a). PBPs are positioned in lymph of sensilla trichodea and have high binding affinities with sex pheromone (Steinbrecht et al., 1995; Forstner et al., 2006; Große-Wilde et al., 2007; Forstner et al., 2009; Gu et al., 2013a), while general OBPs binding to plant volatiles are found in either sensilla basiconica or sensilla trichodea, or both (Hekmat-Scafe et al., 1997; Zhang et al., 2001; Wang et al., 2003; Sun et al., 2014). Moreover, some OBPs are expressed in leg, larval antenna, maxillary palp, mouthpart and proboscis (Bohbot and Vogt, 2005; Sengul and Tu, 2010; Sun et al., 2016; Zhu et al., 2016), even in the non-chemosensory organs, such as reproductive organs of male (Sun Y.L. et al., 2012; Ban et al., 2013) and female (Gu et al., 2013b; Zhang Y.N. et al., 2015). The diverse expression pattern indicates that the function of OBPs is more complicated than previously imagined, beyond the chemosensation. In *Drosophila*, OBP49a is expressed in the thecogen cells of the major taste organ labellum, interacts with bitter chemical, and is required for avoiding bitter-tasting compounds (Jeong et al., 2013); besides, OBP10 from *Helicoverpa* species, able to bind an insect repellent and highly enriched in seminal fluid, is delivered to females during mating and is finally located on shell of fertilized eggs (Sun Y.L. et al., 2012). Recent study shows that the mouthparts enriched OBP11 of the alfalfa plant bug exhibits a strong binding ability to non-volatile plant secondary metabolites, suggesting an involvement in feeding behavior (Sun et al., 2016). Members of OBPs that are found in non- olfactory organs are becoming an interesting aspect of function research.

The tea geometrid *Ectropis obliqua* Prout is one destructive defoliator of tea bushes in China, resulting in considerable economic losses. Given the healthy and environmental risks of chemical control against *E. obliqua*, safer alternatives based on insect-behavior-modifying stimuli are developed to manage this pest, such as synthetic pheromone lures and “push-pull” habitat management (Zhang Z. et al., 2015; Yang et al., 2016). Undoubtedly, deep insights into insect chemical communication could contribute to the design of pest repellents or attractants. For instance, in tortricid moth *Epiphyas postvittana*, the monoterpene citral recognized by OR3 elicits the notable repellent activity against the ovipositing female moths (Jordan et al., 2009). In our previous work, the ultrastructure of antennal and tarsal sensilla in *E. obliqua* moths was observed (Ma et al., 2016a). Subsequently, 24 OBP transcripts were identified from legs transcriptome of *E. obliqua* moths, of which EoblOBP6 showed the highest expression based on RPKM metric (Ma et al., 2016b; Zhang et al., 2018). Previously, many studies have documented the unusual distribution of insect OBPs in non-olfactory organs, but their physiological roles remain largely unknown. Here we focus on EoblOBP6, particularly for its high abundance and the dual expression pattern in antennae and legs. In this work, the specific sensillum location of EoblOBP6 is investigated by cellular immunolocalization, and the ligand-binding specificity of EoblOBP6 to host volatiles, non-host plant volatiles, herbivore-induced volatiles, plant secondary metabolites and tastants are further measured.

## Materials and Methods

### Insect Rearing and Tissue Collection

Adult *E. obliqua* were originally collected from the experimental tea plantation of the Tea Research Institute, Chinese Academy of Agricultural Sciences (Hangzhou, Zhejiang, China). The laboratory colony was reared on fresh tea shoots in enclosed nylon mesh cages and maintained in controlled environment of 25 ± 1°C and 70 ± 5% relative humidity under a photoperiod of 14-h light: 10-h dark. After pupation, female and male individuals were kept separately until eclosion. After emergence, moths were supplied with 10% honey solution. Different tissues from *E. obliqua* adults of both sexes including antennae, stylets, heads (without antennae), thoraxes, abdomens, legs and wings were sampled for both RT-PCR and western blot analysis. Three biological pools were prepared, and all samples were frozen immediately and stored in -80°C.

### RNA Extraction and cDNA Synthesis

Total RNA of each sample was extracted by using Trizol reagent (Invitrogen, Carlsbad, CA, United States). The integrity and purity of extracted total RNA was examined with 1.0% agarose electrophoresis, and RNA quantity was determined using a spectrophotometer NanoDropTM (NanoDrop Inc., Wilmington, DE, United States). A FastQuant RT-kit with gDNA Eraser (TianGen, Beijing, China) was employed to synthesize the first-strand cDNA using 2 μg RNA.

### qRT-PCR Analysis and RT-PCR Verification

The expression profiles of EoblOBP6 (Accession No. ALS03854.1) in different tissues were determined by RT-PCR (Supplementary Table S1). Each PCR reaction contained 200 ng cDNA template, performed by Taq Master Mix (CWBIO, Beijing, China) under a general 3 step amplification by 33 cycles of 94°C for 20 s, 58°C for 20 s, 72°C for 40 s. PCR products were checked by electrophoresis and further confirmed by sequencing. The β-actin gene (Accession No. KT860051) was served as an endogenous control. Each reaction was performed three times with different biological samples.

The relative expressions of EoblOBP6 among tissues were measured by qRT-PCR on a Bio-Rad CFX96 touch real-time PCR detection system. Two reference gene, β-actin and GAPDH (Accession No. KT991373), were employed to normalize the target EoblOBP6 expression and to rectify the sample-to-sample discrepancy. To determine the amplification efficiencies of the reference and target genes, the efficiency of each primer pair was measured by constructing a standard curve with serial template dilutions. The standard curves created regression line with slopes ranging from -3.4 to -3.3, and the amplification efficiency of target gene was approximate to that of the reference genes. The qRT-PCR reaction using SuperReal PreMix Plus (TianGen, Beijing, China) was performed as previously reported (Ma et al., 2016b). The relative transcript level was calculated by the comparative 2^-ΔΔCT^ method.

### Recombinant Protein Expression and Purification

The open reading frame of EoblOBP6 was amplified for the construction of recombinant expression vector. PCR reaction was performed as follows: initial denaturation at 95°C for 2 min, followed by 35 cycles of 94°C for 20 s, 58°C for 30 s and 72°C for 30 s, and a final elongation step at 72°C for 10 min. The correct product confirmed by sequencing was subcloned into the bacterial expression vector pET32a(+). The recombinant plasmid was then transformed into *Escherichia coli* BL21 (DE3) cells. The recombinant protein was induced at 37°C for 6 h with 1 mM isopropyl ß-D-1-thiogalactopyranoside (IPTG). The protein was purified by two rounds of Ni^2+^ ion affinity chromatography with gradient concentration imidazole washing, and the His-tag was excised using recombinant enterokinase (Novagen, Madison, WI, United States). The purified protein was desalted through extensive dialysis, and the size and purity of the recombinant protein were confirmed by 15% SDS-PAGE analysis.

### Western Blot Analysis

The polyclonal antibody against EoblOBP6 was produced by injecting adult rabbits subcutaneously with the purified recombinant protein. The immunized rabbits were reared individually in comfortable cages, and all procedures were operated conforming to the ethical guidelines to minimize pain and discomfort to the animals. The serum was purified using a MAb trap kit (GE Healthcare).

The tissue was homogenized in lysis buffer (8 M urea, 4% chaps, 40 mM Tris–HCl, 5 mM EDTA, 1 mM PMSF and 10 mM DTT, pH 8.0) containing a mixture of protease inhibitors (Roche, Switzerland). Crude protein extracts from adult tissues, including female legs, male legs, female antennae and male antennae, were quantified by a Bio-Rad protein assay with bovine serum albumin (BSA) as standard, and then diluted to obtain equal amounts of the total proteins. After separation by 15% (w/v) SDS-PAGE, samples were transferred onto nitrocellulose (NC) membrane blotting filters at 100 V for 1 h at 4°C. Membranes were then blocked with 5% (w/v) skimmed milk in PBST at 4°C overnight. After washing thrice with PBST, the blocked the membrane was incubated with β-actin antibody (1:2000 dilution) and EoblOBP6 antibody (1:4000 dilution) for 1 h at room temperature, separately. After three washes with PBST, the membrane was incubated for 1 h at room temperature with goat anti-rabbit IgG HRP-linked secondary antibody (Sigma, St. Louis, MO, United States) at 1:10,000 dilution with PBST. The immunoreactivity was visualized using an enhanced electrochemiluminescence detection kit (TransGen, Beijing, China) and photographed by Image Quant LAS4000 mini (GE-Healthcare, Germany). Additionally, western blot analysis was also performed to examine the specificity of the antibody using the purified EoblOBP6 protein.

### Immunocytochemical Localization

The foreleg tarsus of female adult, and intact antennae detached from male and female adults were prefixed in a mixture of paraformaldehyde (4%) and glutaraldehyde (2%) in 0.1 M PBS (pH 7.4) for 24 h at room temperature, dehydrated in an ethanol series, and then embedded in Luria-Bertani white resin (Taab, Aldermaston, United Kingdom) for polymerization at 60°C. Ultrathin sections (60 nm) were cut by a diamond knife on a Reichert Ultracut ultramicrotome (Reichert Co., Vienna, Austria). For immunostaining, the grids were floated in droplets of PBS (containing 50 mM glycine), followed by PBGT (PBS containing 0.2% gelatine, 1% bovine serum albumin, and 0.02% Tween-20). The grids were then incubated with EoblOBP6 antiserum (diluted at 1:3000) at 4°C overnight. After rinsing six times in PBGT, the grids were incubated with secondary antibody (anti-rabbit IgG) coupled with 10 nm colloidal gold granules (Sigma) (diluted at 1:20) for 90 min at room temperature. The grids were then transferred to silver intensification and stained with 2% uranyl acetate to increase the contrast. Finally, sections were observed with HITACHI H-7500 TEM (Hitachi Ltd). The serum supernatant from an uninjected rabbit was used as the negative control.

### Fluorescence Competitive Binding Assays

For the ligand binding assays, the tested compounds, including terpenoids, tea volatiles, herbivore-induced plant volatiles and non-volatile tastants, were selected according to the previously reported isolation from the *E. obliqua* host plant and non-host plant (Sun X.L. et al., 2014; Zhang Z. et al., 2015). Fluorescence binding assays were performed on a fluorescence spectrophotometer F-380 (Tianjin, China) with a 1 cm light path quartz cuvette and 10 nm slits for excitation and emission. The excitation wavelength was set at 337 nm, and the emission spectrum was recorded between 390 and 500 nm. Both the fluorescent probe *N*-phenyl-1-naphthylamine (1-NPN) and the tested chemicals were dissolved in methanol in preparation for 1 mM stock solution. To determine the dissociation constant of EoblOBP6 with 1-NPN, 2 μM protein solution in 50 mM Tris-HCl (pH 7.4) was titrated with aliquots of 1 mM 1-NPN solution to final concentrations ranging from 1 to 16 μM. Then the affinities of ligands were tested by competitive binding assays through titrating the chemical competitor from 2 to 30 μM into the 1-NPN and EoblOBP6 mixed solution (both at 2 μM). The fluorescence intensities at the maximum fluorescence emission were plotted against the free ligand concentration to determine the binding constants. The bound ligand was evaluated from the fluorescence intensity in the assumption of the protein was 100% dynamic, with a stoichiometry of 1:1 (protein: ligand) at saturation. The binding curves were linearized using Scatchard Plot. The dissociation constants of competitors were calculated from the corresponding IC_50_ values following the equation: Ki = (IC_50_)/(1+(1-NPN)/*K*_1-NPN_), where (1-NPN) is the free concentration of 1-NPN and *K*_1-NPN_ is the dissociation constant of the protein/1-NPN complex.

### Homology Modeling and Phylogenetic Analysis

The SWISS-MODEL workspace^1^ (Biasini et al., 2014) was employed to search for the most suitable template to build the 3D structure. Because of the high global quality estimation score (GMQE) with EoblOBP6, the template structure of *Bombyx Mori* GOBP2 was selected for the homology modeling by means of automatic mode. A Ramachandran plot was employed to evaluate the rationality of the established model. The secondary structure was predicted by ESPript 3.0 program (Robert and Gouet, 2014) based on the constructed 3D model and the aligned sequences. The 158 OBP sequences from Lepidoptera species were selected for elucidating the evolutionary history (Supplementary Table S3). The phylogenetic tree was constructed by MEGA 6.0 using the Neighbor-joining mode with a p-distance model and a pairwise deletion of gaps. Bootstrap support was assessed by a boot strap procedure based on 1000 replicates.

## Results

### Tissue Expression Pattern of EoblOBP6

**FIGURE 1:**
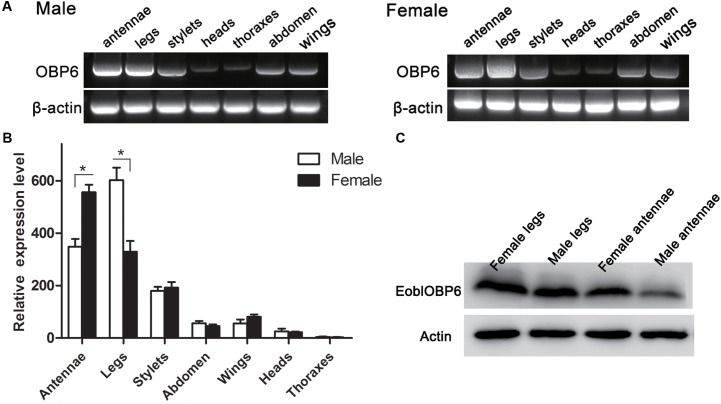
The tissue distributions of *E. obliqua* odorant-binding protein 6 (EoblOBP6). The detection of EoblOBP6 expression in various tissues by RT-PCR **(A)** and qRT-PCR analysis **(B)**. **(C)** Western blot analysis of EoblOBP6 expression in total protein extracts of adult antennae and legs in both sexes. In qRT-PCR, the internal control β-actin and GAPDH were used to normalize transcript levels in each sample. The relative fold-changes representing the relative expression levels were calculated relative to the transcript level in the thoraxes. The standard error is represented by the error bar, and asterisk indicates significant differences between groups (*P* < 0.05) by Student’ *t*-test.

The RT-PCR results indicated that EoblOBP6 was clearly detected in both antennae and legs of adults in both sexes, whereas a plain band was also observed in stylets, abdomen and wings in both sexes (**Figure 1A**). The relative expression was further confirmed by qRT-PCR measurement. The results revealed that EoblOBP6 transcripts were abundantly transcribed in tissues of antennae and legs, followed by stylets. EoblOBP6 was weakly expressed in wings, abdomen and heads. Besides, higher transcripts abundance was detected in female antennae than that in male antennae (**Figure 1B**). Meanwhile, western blot analysis confirmed EoblOBP6 protein was distributed in adult antennae and legs (**Figure 1C**).

### Expression and Purification of EoblOBP6

**FIGURE 2:**
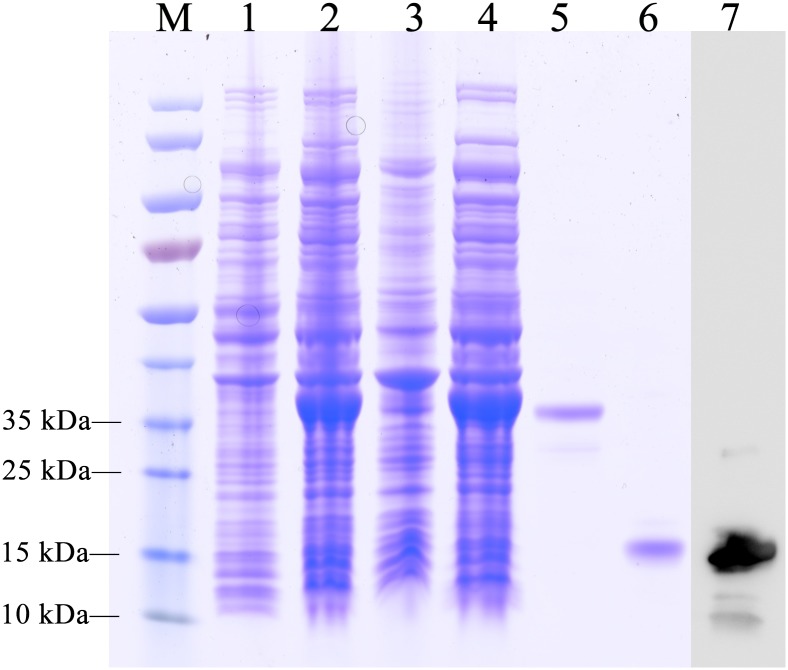
SDS-PAGE analysis of the purification of recombinant *E. obliqua* odorant-binding protein 6 (EoblOBP6). M, protein molecular weight marker; Lane 1, non-induced PET/EoblOBP6; Lane 2, induced PET/EoblOBP6; Lane 3, inclusion body of disrupted IPTG induced cells; Lane 4, supernatant of PET/EoblOBP6; Lane 5, purified EoblOBP6 with His-tag. Lane 6, purified EoblOBP6 cleaved His-tag by rTEV protease; Lane 7, western blot analysis of the purified EoblOBP6 using polyclonal rabbit antiserum.

The recombinant protein of EoblOBP6 was successfully expressed in a bacterial expression system and purified twice using Ni^2+^ ion affinity chromatography, followed by excision of the His-tag with enterokinase. The SDS-PAGE analysis showed the highly purified protein as a single band with the molecular weight of approximately 14 kDa (**Figure 2**), consistent with the predicted molecular mass.

### Specific Localization of EoblOBP6 in Sensilla Chaetica

**FIGURE 3:**
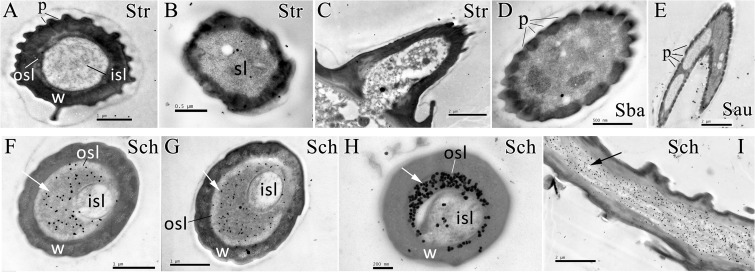
Immunocytochemical localization of EoblOBP6 in different antennal sensilla from *E. obliqua* adult. The immunolocalization of EoblOBP6 in types of sensilla was determined in both sexes, and no significant difference was observed. Black spots (arrow indicates the location) represent the immunostained EoblOBP6 protein. EoblOBP6 was not stained in either sensillum lumen (sl) or cuticle in sensilla trichodea (Str) (cross sections **A**,**B** and longitudinal sections **C**). No specific staining was observed in the sensillum lymph of sensilla basiconica (Sba) or sensilla auricillica (Sau) **(D,E)**. EoblOBP6 was predominantly labeled in the outer sensillum lymph (osl) of sensilla chaetica (Sch), which is not innervated by sensory neurons; the inner sensillum lymph (isl) where neuronal dendrites reside showed few unspecific gold grains (cross sections **F**,**G**,**H** and longitudinal sections **I**). w, sensillum wall; p, pores.

The polyclonal antiserum against recombinant EoblOBP6 protein was prepared to investigate the cellular immunolocalization in distinct sensilla of adult antennae and foreleg tarsomere according to the previous elucidation of sensillum ultrastructures (Ma et al., 2016a). First, the specificity of antiserum was confirmed by western blot analysis, and EoblOBP6 antibody could reacted specifically with EoblOBP6 protein (**Figure 2**). The immunostaining of EoblOBP6 in antennal sensilla indicated that EoblOBP6 was predominantly labeled in the large outer sensillum lymph of sensilla chaetica, which is not innervated by sensory neurons. Although the crescent-shaped outer sensillum lumen was heavily labeled, the inner dendritic cytoplasm and the cuticle of the hair wall showed more than few unspecific gold spots (**Figures 3F–I**). Both crosswise and longitudinal sections indicated that the sensillum lymph of sensilla chaetica was intensely stained by the anti-EoblOBP6 antiserum, and the fierce immunolabeling was detected in the top sections. No obvious staining was observed in either sensilla trichodea or sensilla basiconica, neither in sensilla auricillica (**Figures 3A–E**).

**FIGURE 4:**
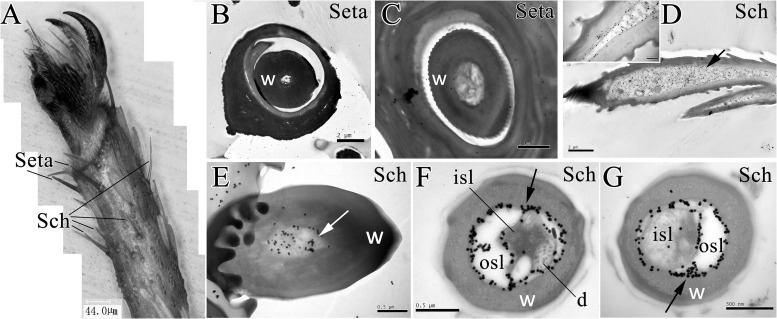
Immunolabeling of EoblOBP6 in types of sensilla present on *E. obliqua* moth fifth tarsomere. Black spots (arrow indicates the location) represent the immunostained EoblOBP6 protein. The sensilla chaetica (Sch) and the mechano-sensitive setae were observed on foreleg fifth tarsomere **(A)**. EoblOBP6 was not stained in either wall or lumen in seta **(B,C)**. Longitudinal sections of sensilla chaetica revealed the strong labeling of EoblOBP6 in sensilla cavity beneath the cuticle **(D)**. Basic section of sensilla chaetica indicated the staining of EoblOBP6 in sensilla lumen but not the sensilla wall **(E)**. Heavy labeling of anti-EoblOBP6 antibody (black spots) was specifically present in the crescent-shaped outer sensillum lymph (osl) which are devoid of the receptor-cell dendrites; the innervated inner sensillum lumen (isl) showed few unspecific gold grains **(F,G)**. w, sensillum wall.

Moreover, the microscopy of *E. obliqua* moths revealed the distribution of setae and sensilla chaetica in the ventral side of foreleg fifth tarsomere (**Figure 4A**). The seta had a thick sensillum wall with no pores. Results of the immunostaining showed that anti-EoblOBP6 antibody specifically labeled the outer sensillum lymph of sensilla chaetica, which housed the receptor cell dendrites. And the fierce immunolabeling was observed to encircle the inner sensillum lumen. However, no obvious staining was detected in the inner sensillum lumen where several neuronal dendrites reside (**Figure 4**).

### Ligand Binding Assays of EoblOBP6

**FIGURE 5:**
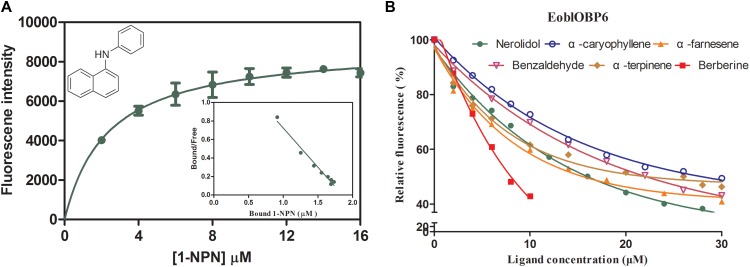
Fluorescence competitive binding assay of *E. obliqua* odorant-binding protein 6 (EoblOBP6). **(A)** Binding curve and relative Scatchard plot for 1-NPN and EoblOBP6. **(B)** Competitive binding curves of the active volatiles and tastants to EoblOBP6.

In preparation for the ligand binding assay, the binding affinity of the fluorescent probe 1-NPN with the purified EoblOBP6 was first measured (**Figure 5**). Results revealed that EoblOBP6 was capable of binding 1-NPN with binding affinity of 2.70 ± 0.24 μM. Subsequently, the binding properties of EoblOBP6 to the selected host compounds from different functional groups were measured, and the results indicated that EoblOBP6 displayed a relatively narrow binding spectrum (**Figure 5** and **Table 1**). Of the 52 tested compounds, only five odorants and one tastant exhibited strong binding abilities to EoblOBP6 (Supplementary Table S2). For the non-host volatiles, two terpenoids, α-caryophyllene and α-terpinene, showed binding affinity to EoblOBP6, with dissociation constants of 15.55 and 18.31 μM, respectively. Besides, the majority of host volatiles, including (Z)-3-hexenol, decanal, 1-hexanol and hexyl acetate, could hardly bind to the recombinant protein, except for benzaldehyde (Ki = 15.08 μM). Interestingly, nerolidol and α-farnesene, volatiles dramatically induced by the herbivore infestation (Sun X.L. et al., 2014), exhibited high binding affinities with EoblOBP6 of 10.87 and 11.02 μM, respectively. For the non-volatile tastants, EoblOBP6 could only bind strongly to berberine (**Table 2**).

**Table 1:** The ligands used for binding analysis to EoblOBP6.

Ligand	CAS Number	EoblOBP6	
		
		IC_50_ (μM)	K_i_ (μM)
Terpenoids		
Limonene	5989–27-5	u.d.	u.d.
β-Myrcene	123-35-3	u.d.	u.d.
*cis*-Verbenol	18881-04-4	u.d.	u.d.
(-)-β-Pinene	18172-67-3	u.d.	u.d.
β-Ionone	14901-07-6	u.d.	u.d.
(+)-Camphene	5794-03-6	u.d.	u.d.
α-Caryophyllene	6753-98-6	22.36 ± 0.27	15.55 ± 0.19
β-Caryophyllene	87-44-5	u.d.	u.d.
β-Ocimene	13877-91-3	u.d.	u.d.
α-Pinene	7785-70-8	u.d.	u.d.
Camphor	464-48-2	u.d.	u.d.
α-Terpineol	8000-41-7	u.d.	u.d.
Verbenone	1196-01-6	u.d.	u.d.
γ-Terpinene	99-85-4	u.d.	u.d.
α-Terpinene	99-86-5	26.24 ± 0.53	18.31 ± 0.37
Intact tea plant volatiles		
(Z)-3-hexenol	928-96-1	u.d.	u.d.
Hexyl acetate	142-92-7	u.d.	u.d.
Nonanal	124-19-6	u.d.	u.d.
Methyl salicylate	119-36-8	u.d.	u.d.
Indole	120-72-9	u.d.	u.d.
Decanal	112-31-2	u.d.	u.d.
1-Hexanol	111-27-3	u.d.	u.d.
Benzaldehyde	100-52-7	21.62 ± 0.84	15.08 ± 0.58
Volatiles from tea plants infested by *E. obliqua* larvae		
(E)-2-hexenal	6728-26-3	u.d.	u.d.
(Z)-3-hexenyl butyrate	16491-36-4	u.d.	u.d.
(Z)-3-hexenyl hexanoate	31501-11-8	u.d.	u.d.
(Z)-3-hexenal	6789-80-6	u.d.	u.d.
Benzyl nitrile	140-29-4	u.d.	u.d.
Benzyl alcohol	100-51-6	u.d.	u.d.
Nerolidol	7212-44-4	15.55 ± 0.76	10.87 ± 0.53
α-Farnesene	502-61-4	15.75 ± 0.42	11.02 ± 0.29
(Z)-3-hexenyl acetate	3681-71-8	u.d.	u.d.
Linalool	78-70-6	u.d.	u.d.


**Table 2:** The non-volatile ligands used for binding analysis to EoblOBP6.

Ligand			OBP6	
	
Tastant	CAS number	Taste perception	IC_50_ (μM)	K_i_ (μM)
D(-)-Salicin	138-52-3	Bitter	u.d.	u.d.
Atropine	51-55-8	Bitter	u.d.	u.d.
Berberine	141433-60-5	Bitter	7.94 ± 0.61	5.55 ± 0.87
Caffeine	21399	Bitter	u.d.	u.d.
Catechin	154-23-4	Bitter	u.d.	u.d.
D-Sucrose	57-50-1	Sweet	u.d.	u.d.
Trehalose	6138-23-4	Sweet	u.d.	u.d.
D-Glucose	50-99-7	Sweet	u.d.	u.d.
D-Xylose	58-86-6	Sweet	u.d.	u.d.
L-Alanine	56-41-7	Sweet	u.d.	u.d.
L-Histidine	71-00-1	Bitter	u.d.	u.d.
Theophylline	58-55-9	Bitter	u.d.	u.d.


### Homology Modeling

**FIGURE 6:**
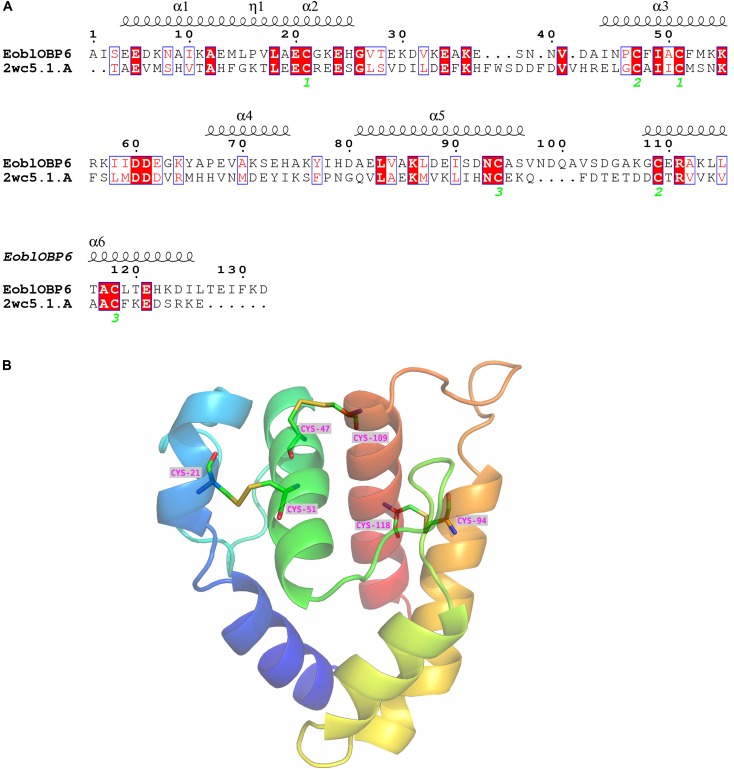
Secondary and 3D structures of *E. obliqua* odorant-binding protein 6 (EoblOBP6). **(A)** Sequence alignment of EoblOBP6 with GOBP1 from *Bombyx Mori* (template library identity: 2wc5.1). α-helices are displayed as squiggles. Identical residues are highlighted in white letters with a red background. Residues with similar physicochemical properties are shown in red letters with a blue frame. The conserved cysteines are labeled in green italic number. The signal peptides are removed. **(B)** The predicted 3D structure of EoblOBP6. Six α-helices, N-terminus and C-terminus are displayed.

The SWISS-MODEL workspace was employed to search for the structural template. The GOBP1 from *Bombyx Mori* (template library identity: 2wc5.1) shared 31% homology with EoblOBP6 and gained global quality estimation score (GMQE) of 0.59, and thus was chosen as the template for homology modeling. The result of Ramachandran plot showed that 94.3% of the residues were in preferred regions, 4.9% of the residues were in the allowed region and 1 residue was identified as an outlier (Supplementary Figure S2), suggesting that the predicted model is generally reliable. The predicted 3D structure of EoblOBP6 was composed of six α-helices between residues Glu4-Leu15 (α1), Ala19-His25 (α2), Ile44-Lys54 (α3), Pro67-His74 (α4), Ala81-Ser96 (α5) and Gly108-Ile125 (α6), forming an α-helix-enriched globular protein. Three pairs of disulphide bridges connecting Cys21 in α2 and Cys51 in α3, Cys47 in α3 and Cys109 in α6, Cys94 in α5 and Cys118 in α6 contributed to the stability of the tertiary structure and the formation of α-helixes (**Figure 6**).

To deduce the evolutionary relationships and underlying functions, 158 lepidopteran OBPs from six species were chosen for phylogenetic tree construction (Supplementary Figure S1). The results revealed a divergent OBP repertoire, and EoblOBP6, EoblOBP22, HarmOBP4 and SexiOBP3 clustered into a same clade. Multiple alignment showed EoblOBP6 shared 46, 40, 32% identity to EoblOBP22, HarmOBP4 and SexiOBP3, separately (Supplementary Figure S1). Overall, these results indicated a specific evolutionary status of EoblOBP6 different from the other lepidopteran OBPs.

## Discussion

In present study, we reveal that EoblOBP6 possesses a dual expression pattern in adult antennae and legs in both sexes, and it is predominantly expressed in the outer sensillum lymph of the uniporous sensilla chaetica. This unique distribution pattern arouses great interest owing to that sensillum chaetica is generally considered as the typical mechano-sensitive sensillum. Several studies have documented the expression of OBPs beyond the olfactory organs, and their physiological functions would be more complicated. Herein we intend to investigate the potential involvement of EoblOBP6 in gustatory and olfactory sensation.

The tea geometrid, *Ectropis obliqua*, is one lepidopteran pest feeding exclusively on tea leaves and tender buds. The female moths possess a remarkable capability to locate suitable host plants which is fundamental to the survival of their offspring, because the young larvae cannot easily forage for alternative hosts (Ryuda et al., 2013). Host plant selection by herbivorous insects involves searching, landing, contact evaluation, and a final decision for acceptance or rejection (Schoonhoven et al., 2005). Contact chemosensilla play a dominant part in detecting phytochemical compounds after landing on plant, which allow insects to perceive the compounds on/in the surface of leaves and flowers (Chapman, 2003; Calas et al., 2007; Newland and Yates, 2008; Zhang et al., 2010). Typically, insect contact chemoreceptors are derived from mechanosensory bristles and are mainly scattered on tarsi, ovipositor, mouthparts, and antennae (Chapman, 2003). Previous study in *E. obliqua* has documented that the arrangement of uniporous sensilla chaetica comprised the majority of chemosensilla in tarsi, and are presumed to be responsible for gustatory cognition (Ma et al., 2016b). Actually, many studies have documented that the arrangement of lepidopteran tarsal chemosensilla are responsible for tastant recognition. In butterflies, including *Papilio xuthus*, *Papilio polytes*, and *Heliconius melpomene*, female butterflies recognize the oviposition stimulants by the contact chemosensilla distributed on the ventral side of their foreleg tarsus (Nakayama et al., 2003; Briscoe et al., 2013); while in moths of *H. armigera*, *Mnesampela privata*, and *Lobesia botrana*, chemosensilla sensitive to sugars or amino acids are situated on the ventral surface of the fifth tarsomere (Calas et al., 2006, 2009; Zhang et al., 2010). In general, contact chemoreceptors respond to chemicals of low-volatility, and have a single pore at the distal tip through which chemicals gain access to several sensory neurons.

Our cellular immunolocalization reveals that EoblOBP6 is strongly labeled in the outer sensillum lumen of the contact sensilla chaetica in fifth tarsomere. This remarkable localization pattern suggests EoblOBP6 may function as a carrier to enable the hydrophobic molecules from the outer sensillum-lymph cavity to reach the dendritic membranes in the inner cavity. Moreover, this cellular localization pattern is consistent with the report of the putative OBP PBPRP2 in *Drosophila* that PBPRP2 is expressed in the outer sensilla lumen of taste sensilla, rather than the lumen where the dendrites of the gustatory neurons reside (Shanbhag et al., 2001). It is commonly accepted that sensilla trichodea is sensitive to pheromone, and the non-labeling of EoblOBP6 in sensilla trichodea may due to the absence of EoblOBP6 in pheromone detection. Recent studies have proposed that OBPs expressed in gustatory organs get involved in gustatory coding. In *Drosophila*, OBP49a enriched in labella is indispensable for perceiving the bitter substances, and OBP49a specifically interacted with bitter chemicals, including berberine, denatonium and quinine (Jeong et al., 2013); two OBPs encoded by OBP57e and OBP57d expressed in chemosensory hairs of tarsus are implicated in taste perception as well as the host–plant preference (Matsuo et al., 2007). Actually, the non-volatile plant metabolites are comparable to odors in the way that they are both small hydrophobic molecules, therefore, it is reasonable to conclude that OBPs act as carrier of such type of poorly water-soluble molecule to gustatory receptors, similar to their performance in olfaction. Our results from the ligand binding assay reveal that EoblOBP6 specifically binds to the alkaloid berberine, which is an aversive bitter stimuli to insect (Pontes et al., 2014). In *Drosophila*, OBP28a abundant in proboscis functions as a transporter of bitter tastants to gustatory receptors, modulating the sugar intake in response to bitter tastants (Swarup et al., 2014). Yet unfortunately, there is no direct evidence supporting that the sensilla chaetica of *E. obliqua* respond to bitter substances. Generally, the presence of bitter compounds, an indication of toxicity, is reported by taste organs. Evaluation of these tastants informs the decision as to whether to accept a host plant as food source or oviposition site, and we presume the underlying participation of EoblOBP6 in this process. In such a scenario, EoblOBP6 present in sensilla chaetica may act as a carrier for hydrophobic bitter compounds.

The tissue-biased distributions of OBPs in insect are indicative of biological function. Results from both qRT-PCR and western blot analysis indicate that EoblOBP6 possesses a dual expression pattern in adult antennae and legs from both sexes. In general, an antenna-abundant expression correlates tightly with olfactory sensation, while the abundance in gustatory organs indicates an involvement in taste detection. The fluorescence competition assay provides further insight into the physiological roles of EoblOBP6. The results show that EoblOBP6 displays a strong binding to nerolidol and α-farnesene, both of which are tea plant volatiles dramatically induced by herbivore infestation. These herbivore-associated plant volatiles are closely associated with the host-search behavior of herbivores. Actually, female *E. obliqua* moths are more attracted by the infested tea plants and preferentially oviposit on these plants, in order to reduce the predation by the natural enemies (Sun X.L. et al., 2014). Besides, benzaldehyde emitted from the intact tea leaves has a relative high binding affinity with EoblOBP6 (Maeda et al., 2006); α-terpinene, a type of terpenoid which is mainly emitted from aromatic plants and elicits strong electrophysiological responses from the antennae of *E. obliqua* (Zhang Z. et al., 2015), shows binding affinity to EoblOBP6. Overall, our results propose that EoblOBP6 is a general OBP that selectively binds to odors of host plant source and may play an important part in host location of female *E. obliqua* moths.

Taken together, this study reports the identification of EoblOBP6 expressed in sensilla chaetica of both antennae and tarsus, and EoblOBP6 preferentially binds to the herbivore-induced plant volatiles, host plant volatiles and plant secondary compound. These results indicate the potential involvement of EoblOBP6 in olfactory and gustatory coding, playing a functional role in host location. Given the great economic impact of *E. obliqua*, a deep insight into their chemosensory system would accelerate the development of insect-behavior-modifying stimuli. Further investigations by RNAi or CRISPR/Cas9 editing to establish the EoblOBP6-targeted mutagenesis would be performed in functional study.

## Author Contributions

The experimental plan conceived and designed by LM and WZ. The experiments performed by LM, WZ, and ZL. The data processed and analyzed by LM, WZ, and ZL. Wrote and edited the manuscripts by LM, WZ, XC, ZL, YZ, and ZC.

## Conflict of Interest Statement

The authors declare that the research was conducted in the absence of any commercial or financial relationships that could be construed as a potential conflict of interest. The reviewer TZ declared a shared affiliation, with no collaboration, with one of the authors, YZ, to the handling Editor.

## Footnotes

1 https://swissmodel.expasy.org/
