# Supplementary material for: The Odorant Binding Protein 6 Expressed in Sensilla Chaetica Displays Preferential Binding Affinity to Host Plants Volatiles in Ectropis obliqua
Source: Front Physiol. 2018 May 17;9:534. doi: 10.3389/fphys.2018.00534 (PMC5967201; doi:10.3389/fphys.2018.00534)
Supplement: Supplementary file 1 [file Table_1.DOCX]

The odorant binding protein 6 expressed in sensilla chaetica displays preferential binding affinity to host plants volatiles in *Ectropis obliqua*

Long Ma^1†^, Zhaoqun Li^2†^, Wanna Zhang^4^, Xiaoming Cai^2^, Zongxiu Luo^2^, Yongjun Zhang^3^, Zongmao Chen^2^

1. Jiangxi Key Laboratory of Bioprocess Engineering, College of Life Sciences, Jiangxi Science & Technology Normal University, Nanchang, China.

2. Tea Research Institute, Chinese Academy of Agricultural Sciences, Hangzhou.

3. State Key Laboratory for Biology of Plant Diseases and Insect Pests, Institute of Plant Protection, Chinese Academy of Agricultural Sciences, Beijing, China.

4. Institute of Entomology, Jiangxi Agricultural University, Nanchang, China.

† These authors contributed equally to this work.

Correspondence and requests for materials should be addressed to Yongjun Zhang (yjzhang@ippcaas.cn) and Zongmao Chen ([zmchen2006@163.com](mailto:zmchen2006@163.com))

**Figure S1.** Sequence alignment and phylogeny of EoblOBP6 with other OBPs identified from Lepidoptera species. (A) Neighbor-joining phylogenetic tree was constructed used MEGA 6.0 with a p-distance model and a pairwise deletion of gaps. (B) Sequence alignment was performed using ClustalX 2.1. Conserved Cys were emphasized in red. (C) The percent identity matrix of EoblOBP6 orthologs. Eobl, *E. obliqua*; Slit, *S. litura*; Sexi, *S. exigua*; Bmor, *B. mori*; Aips, *A. ipsilon*; Harm, *H. armigera*.


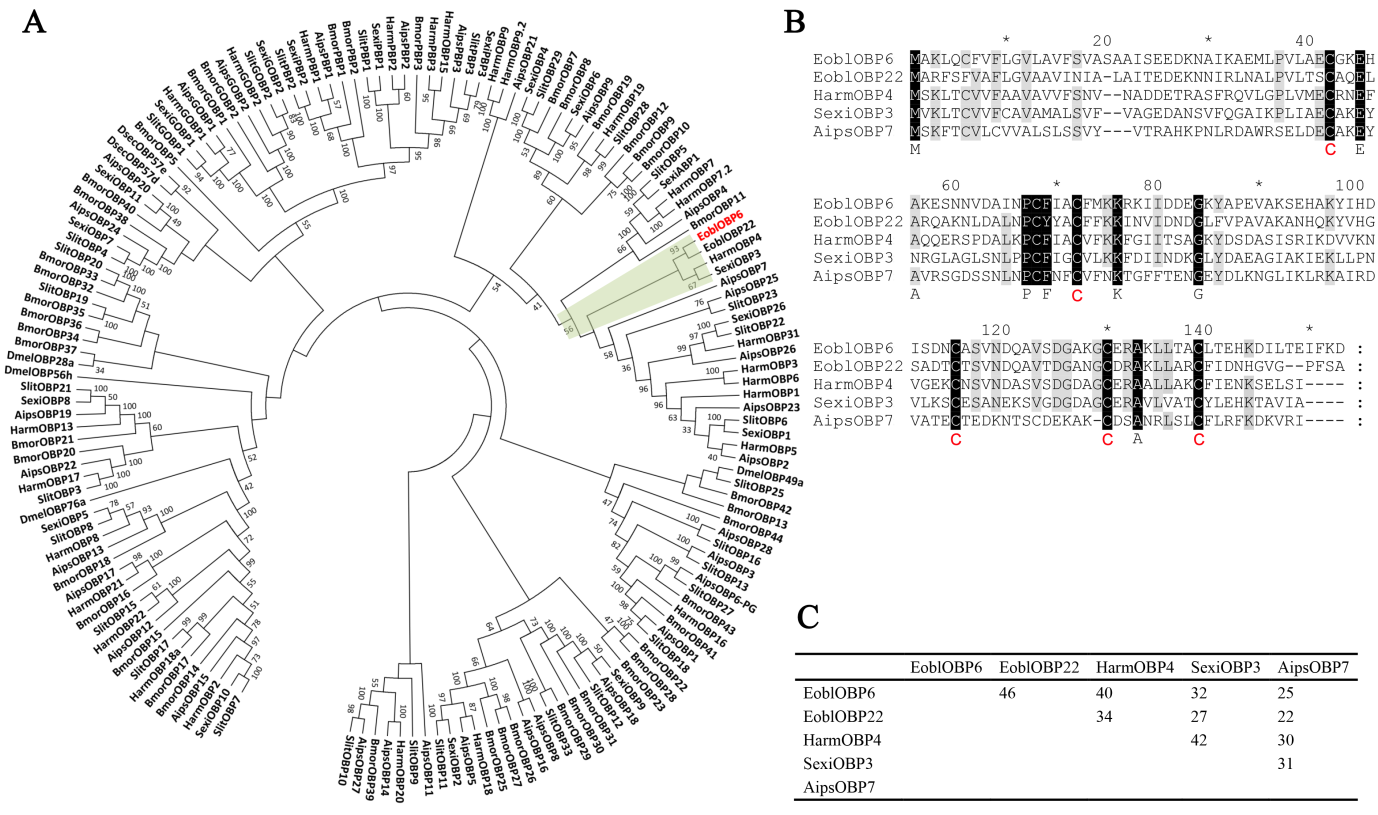


**Figure S2.** Rational evaluation of the established 3D model of EoblOBP6 using a Ramachandran plot. The result showed that 94.3% of the residues were in preferred regions, 4.9% of the residues were in the allowed region and 1 residue was identified as an outlier.


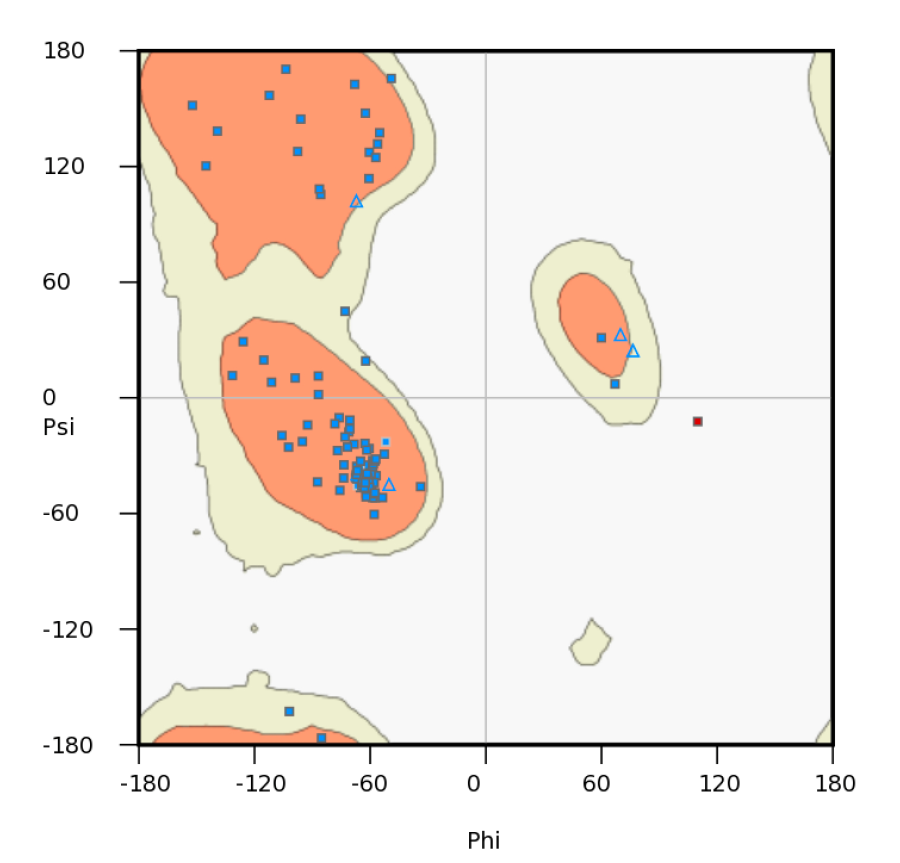


**Table S1.** Primers used in the experiment.

| Primer name | Forward | Reverse |
| --- | --- | --- |
| RT-PCR | | |
| EoblOBP6 | GAGTACTTGCCGTCTTCAGC | CAACTTCGCCCTCTCACAAC |
| ß-actin | TCTTCCAGCCCTCATTCCTG | GGGCTGTGATTTCCTTCTGC |
| qRT-PCR | | |
| EoblOBP6 | CGAGGTGGCTAAATCGGAAC | TCCTTGTGCTCAGTCAGACA |
| ß-actin | TCTTCCAGCCCTCATTCCTG | GGGCTGTGATTTCCTTCTGC |
| GAPDH | AGAACATCATTCCAGCGTCC | CCTCAGCGGCTTCCTTG |

**Table S2.** Structural formula of the active ligands of EoblOBP6.

| Ligand | CAS Number | Structural formula |
| --- | --- | --- |
| α-Caryophyllene | 6753-98-6 | 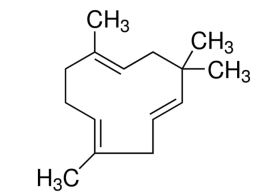 |
| α-Terpinene | 99-86-5 | 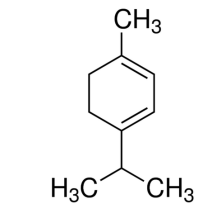 |
| Benzaldehyde | 100-52-7 | 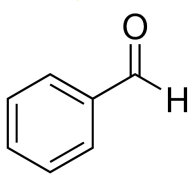 |
| Nerolidol | 7212-44-4 | 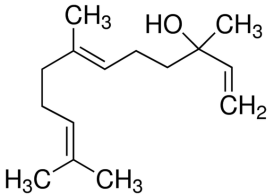 |
| α-Farnesene | 7212-44-4 | 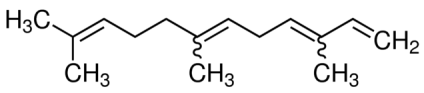 |
| Berberine | 141433-60-5 | 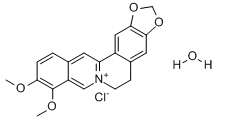 |

**Table S3.** 158 OBP sequences from Lepidoptera species used in the phylogenetic tree construction.

>EoblOBP6

MAKLQCFVFLGVLAVFSVASAAISEEDKNAIKAEMLPVLAECGKEHGVTEKDVKEAKESNNVDAINPCFIACFMKKRKIIDDEGKYAPEVAKSEHAKYIHDAELVAKLDEISDNCASVNDQAVSDGAKGCERAKLLTACLTEHKDILTEIFKD*

>EoblOBP22

MARFSFVAFLGVAAVINIALAITEDEKNNIRLNALPVLTSCAQELGIKMEDVVAARQAKNLDALNPCYYACFFKKINVIDNDGLFVPAVAKANHQKYVHGADDLARLSASADTCTSVNDQAVTDGANGCDRAKLLARCFIDNHGVGPFSA*

>DmelOBP28a

MQSTPIILVAIVLLGAALVRAFDEKEALAKLMESAESCMPEVGATDADLQEMVKKQPASTYAGKCLRACVMKNIGILDANGKLDTEAGHEKAKQYTGNDPAKLKIALEIGDTCAAITVPDDHCEAAEAYGTCFRGEAKKHGLL

>DmelOBP56h

MKFTLFCIALAAFLSMGQCNPDFRQIMQQCMETNQVTEADLKEFMASGMQSSAKENLKCYTKCLMEKQGHLTNGQFNAQAMLDTLKNVPQIKDKMDEISSGVNACKDIKGTNXCDTAFKVTMCLKEHKAIPGHH

>DmelOBP76a

MKHWKRRSSAVFAIVLQVLVLLLPDPAVAMTMEQFLTSLDMIRSGCAPKFKLKTEDLDRLRVGDFNFPPSQDLMCYTKCVSLMAGTVNKKGEFNAPKALAQLPHLVPPEMMEMSRKSVEACRDTHKQFKESCERVYQTAKCFSENADGQFMWP

>DsecOBP57d

MPGKMSLRFLPHLACIIFILEIQFRNSECNDPCPHNEGIDEDIAEAILSDWPANVDLTSVKRSHKCYVTCILQYYNIVSTSGEIFLDKYYDTGVIDEFAVAPKINRCRYEFRMETDYCSRIFAIFNCLRQEILAN

>DsecOBP57e

MLDRLTLYLLINFLCANVLAYTSVFNPCVSQNELSEYEANQVMEKWPDPPIDRAYKCFLTCVLLDLGLIDERGNVQIDKYMKSGVVDWQWVAIELVTCRIEFSDERDLCELSYGIFNCFKDVKLAAEKNVSISNGK

>DmelOBP49a

MLSKSQLLLLVVGFCLNAAVSADVDCSKRPSFVNPKTCCPMPDFVTAELKQKCIKFDMTPPPPPDGEASGSFESKRRHHHPHPPPCFFSCIFNETGIYQNRKLDEAKLNAYLQEVFEDSSDLQTTATQAFTTCATKVADFEANLPPRPAPSPPPGFPMCPHDAGHLMGCVFRNMMKNCPDSIRNDSQQCTDMKEFFTKCKPPRGPPPSAEDM

>BmorGOBP1

MWKLVVVLTVNLLQGALTDVYVMKDVTLGFGQALEQCREESQLTEEKMEEFFHFWNDDFKFEHRELGCAIQCMSRHFNLLTDSSRMHHENTDKFIKSFPNGEILSQKMIDMIHTCEKKFDSEPDHCWRILRVAECFKDACNKSGLAPSMELILAEFIMESEADK

>BmorGOBP2

MFSFLILVFVASVADSVIGTAEVMSHVTAHFGKTLEECREESGLSVDILDEFKHFWSDDFDVVHRELGCAIICMSNKFSLMDDDVRMHHVNMDEYIKGFPNGQVLAEKMVKLIHNCEKQFDTETDDCTRVVKVAACFKKDSRKEGIAPEVAMIEAVIEKY

>BmorPBP1

MSIQGQIALALMVYMAVGSVDASQEVMKNLSLNFGKALDECKKEMTLTDAINEDFYNFWKEGYEIKNRETGCAIMCLSTKLNMLDPEGNLHHGNAMEFAKKHGADETMAQQLIDIVHGCEKSTPANDDKCIWTLGVATCFKAEIHKLNWAPSMDVAVGEILAEV

>BmorPBP2

MKLQVVLVVLTVEMVCGSRDVMTNLSIQFAKPLEACKKEMGLTETVLKDFYNFWIEDYEFTDRNTGCAILCMSKKLELMDGDYNLHHGKAHEFARKHGADETMAKQLVDLIHGCSQSVATMPDECERTLKVAKCFIAEIHKLKWAPDVELLMAEVLNEVSWKS

>BmorPBP3

MARYNIVVAVLVLGVVGARGSSEAMRHIATGFIRVLDECKQELGLTDHILTDMYHFWKLDYSMMTRETGCAIICMSKKLDLIDGDGKLHHGNAQAYALKHGAATEVAAKLVEVIHGCEKLHESIDDQCSRVLEVAKCFRTGVHELHWAPKLDVIVGEVMTEI

>BmorOBP5

MKQRLRVLLLRFCILQTVLSESGVDVVKNLSLSFARFFLECDEERHFQPEVRLKVMTFWYSESSTWDRDVGCAFLCIFKKMEIDNPQDPSYRTHLELLSFANSEDNKIANQMVEIFYACGENTETDPCLWALEQVKCYKNRINQLGLTPTF

>BmorOBP7

AVTEEELKIEFTKLVMKCTKDHPVDMSELMQLQQLIAPKKTESKCLLACAYKLNGVMTSQGLYNLEHAYKIAEMSKNGDEKRLENGKKVADICVKVNDVEVSDGE KGCERAALIFKCTLENAPKVFKFGSSEYNCQ

>BmorOBP8

MLRVVVICVCFLVIAPYGINASSLDDLKMVYKNVIKECVGDYPITAADLKLIKARQIPNDDIKCVFACAYKKTGMMTEEGMLSVEGIKDMSQKYLSDNPEQLRKSKEFAEACSSVNDQQVSDGTKGCERAALIFKCSTEKITNFGFEL

>BmorOBP9

MLRVVVICVCFLVVAPYGINAVSYEQKIKIRDQLDRAGFECFKDHKITEDDIKNLRANKPATGENVPCFIACVMKKTGVMNDQGVIRKGPVLELAKKVLADDKDIKKLQDYIHSCSHVNSETVHDKGKGCEFAMQAYTCMSANASKFGFNI

>BmorOBP10

MLRVVVICVCFLVIAPYGINAVSDEQKIKIREQIDKSGFECFKDHKITEDDIKNLRARKPATGENVPCFIACVMKKTGVMNDQGVIHTEPVLQLAKKVLTDDKDIKKLQDYIHSCS HVNSKTVHDKGQGCEFAIQTYTCMSANASKFGFDV

>BmorOBP11

MSANSFVVLAFCALAVGVNALTEEQKAEITKSSLPLIAECSKEFSVNQGDIDAAKKLGDPSGLNSCFVGCFMKKAGIINASGLFDVAATIEKSKKYLTSEEDLKAFEKLTETCAPENDKPVSDSDKGCERAKLLLDCFVANKGSFSVFSL

>BmorOBP12

MTSFMVFFVLSVLTLKYSDALTDEQKNKIQSKFIEIGAECIVEHPISIDDINSFKNKKFPSGVNAGCFVACIFNKIGLFDDKGNLSHNSALEKAKGIFNADEEVKNLEEFLNRCAKVNGEAVGDGVKGCERAKLAYNCLIENSLEFGFNIDF

>BmorOBP13

MLKIHVLLCFGMAILYFGSAKAVTPEESKAFEAFAKPVIEQCQKDFGMDKESFAQKNLDEIDECLIACVVEKFGITNDEKIDGDALKALVTKFVGNEEERNKINKIVEECTEDANKSGDGTCNTSTILFLCLLKNGKDLWGF

>BmorOBP14

MSRQQLKNSGKMLKKQCMGKNDVTEEEIGDIEKGKFIEQKNVMCYIACIYQMTQIIKNNKISYEASIKQIDLMYPPELKESAKASAGRCKDVSKKYKDICEASYWTAKCMYEDNPKDFIFA

>BmorOBP15

MFLKNIFIECVLLYFVMLNTSFVNTMTKQQIKNSGKILKKACISKNDVTEDQISDIDKGKFIEDKNVMCYIACVYSMSQVVKNNKFVHDAMVKQVDMMFPTEMRDAVKASIANCRGVAKNYKDICEASFWTAKCMYEFDPANFVFA

>BmorOBP16

MRISFLFLISVTIITFDSVFAMTRAQVKKTMTIMKNQCMPKNGVTEDQVGKIEEGIFLENHNVMCYIACVYKTIQVVKNDRLDKDLISKQIDVLYPQEIRESTKKAVGDCINLQEKYDDWCEGIFRSTKCLYEKDPANFIFP

>BmorOBP17

MTRQQLKNSGKIMKKTCMPKNDVTEEEIGQIEQGKFLEQRNVMCYIACIYTVTQVVKNNKLSYDAVIKQVDVMFPAEMRPAVKAAAENCKDISKTFKDICEASYWTAKCMYDFDPKNFVFP

>BmorOBP18

MILIVIAKFLILISLCETMTMKQIKNTGKMMRKSCQPKNNVDDEKINPINDGVFIEENEVKCYIACIMKMANTMKNGKLNFEAAMKQADLLLPDEMKEPTKEAIVACRKVADSYKDVCDASFHVTKCIYNHNPSVFFFP

>BmorOBP19

MTSAKTDVEIKAWFLGQAVECSKDHPVTTEELRMHKHELPDSKNAKCLMKCVFRKCNWLDSKGMYDINAAYASSTKDFSDDKTKQENANKLFDTCKSVNEENVGDGEEGCDRSLLLAKCLTKAAPQVSIYYS

>BmorOBP20

MAVHIFLILASYMALAAHGQLDDEIAELAAMVRENCADESSVDLNLVEKVNAGTDLATITDGKLKCYIKCTMETAGMMSDGVVDVEAVLSLLPDSLKTKNEASLKKCDTQKGSDDCDTAYLTQICWQAANKADYFLI

>BmorOBP21

MITASLHVIFALLAFVYGGKDKPVLSEEIKEIIQTVHDECVGKTGVSEEDITNCESGIFKEDVKLKCYMFCLLEEAGLVNDDGTVDYEMFTSLIPEEYFDRATKMIFSCKELDTPDKDKCERAFEVHKCSYEKDPDFYFLF

>BmorOBP22

MLKVFVVVVCTLGASQLCAALYTQKVAVSFPKDKTTIVVEAMKSCIAKTGANPNVIEVISSGKVSEDEKFKEFFYCACNDIGVVNPDGHIKVKECIELFPKETQPLVEPVI KNCDKEGVNKYDTLFKYLKCFQETSPVRVTLA

>BmorOBP23

MTSKVLLSCVVLAVLATTVLAEDSRKLVSFAPEVAKKLKVLIQECLNENGLGEDAIEVIRAGEYREDEPFQNLVYCAYKKFGALDENNRIISQVAAASFPKDIDVVTVIESCGKEDGNTPVEQVFKYFKCFQKNSPVRMQLY

>BmorOBP25

MKSVVLICLAFAVFNCGADNVHLNEDEREKANWYTAECGVETGVSTEVINAAKIGKYSKDKAFKKFVLCFFKKSAILNSDGTLNMVVALAKLPSGVNKSEAQSVLEQCKNKTGQDAADKAFAILQCFHKGTKTHILF

>BmorOBP26

MKSVVLICLAFAVFNCGADNVHLAETQKEKAKQYTSECVRESGVSTEAINAAKIGKYSKDKAFKNFVLCFFNKSAIFNSDGTLNMDVALAKLPPGVNKSEAQSVLKQCKNKTGQGAADKAFEIFRCYYKGTKTHILF

>BmorOBP27

MKSVVLICLAFAVFNCGADNVHLTETQKEKAKQYTSECVKESGVSTEVINAAKTGQYSEDKAFKKFVLCFFNKSAILNSDGTLNMDVALAKLPPGVNKSEAQSVLEQCKDKTGQDAADKAFEIFQCYYKGTKTHILF

>BmorOBP28

MLKVFIVTFFAFQLSAIARLQANGCVAVPFPKDKTIIIVEAMKSCIAKTGANPNFIDVIRSGKVSEDEKFKEFYYCTCNDTGFVNPDGHIKVKECIELFPKETQPLVEPVIKNCDKEEGVNKYDTLFKFLKCFQETSPVRVALA

>BmorOBP29

MTGPAAAAVLLALLAAAGQATTGCKNCVILGKEERAMFRSHSDACLAQSRVEPRLLESMMNGELIDDAALRKHVYCVLLSCKMIGKDGKLLKAAILGKLAARPAGRDVTKVLEACAEQPGASPEDVAWNIFRCGYNRKAVLFDYMPAGGASSGNTENHP

>BmorOBP30

MRSFVILLNYGLLCCGQFMAEDYYYDIVTRDPDDLMREKENEVRALRAFQADCAEDVQVKPDLVVNLKSGDWQTEDVSLKKWALCVLMKLGLMTAQGVFKMNEAMSKIPDMNDKIIAEKLIDDCLSLQATTPHDAAWNYIKCHHQKDPEGNFSSLNIF

>BmorOBP31

MKTFIVFVVCVVLAQALTDEQKENLKKHRADCLSETKADEQLVNKLKTGDFKTENEPLKKYALCMLIKSQLMTKDGKFKKDVALAKVPNAEDKLKVEKLIDACLANKGNSPHQTAWNYVKCYHEKDPKHALFL

>BmorOBP32

MYSHKYLNDFTNIPEILIILLSSVALMSYGYNTKLFSHSLGSEPSLSILYARDKKSDKVTNECLMEMYPKNLYKYPLRIDRNDIPCIIHCVLKKFGIISNDGFINIKNYYRRVQAIHRYDPRILISDVGETCAQNINGMNLDHDVCKKAKVFNDCTQLYAISYREPEDW

>BmorOBP33

MYAHDKLSDMIADQCLNEMYPRSKRLEIEESDEPCIIFCVLKKFGIMSPTGVINLEAYRKRVQLPEQLAQRNSINDFGSACLESAEATQHKQDVCKKAKVFNECTHLYKILLK

>BmorOBP34

MEKMILLNVFAVVLPCVLASRTRGSSGTLVDFTDPKVQGHLDALVRMAQSCVIKVRATPKDVRAYFTNSSPVSRSGQCFATCMLEQSDIINHGKVNRDLLVHLAGLVNGKNSRVVRKLNSVSRLCLDSISGMTDRCQLASTYNDCLNENMIEFAFPLDIAEEAVRKMPFHLIQPK

>BmorOBP35

GMSTHVLDFKRNMTECLKEVQNNDKRPIKRLSPKQESPIHGECLIACVLKKNGVIQNGKVNKDNLMALVSKFHAKETKLMKKLEKNLDRCINISVKNHDECSLASQLNDCTNDIMASSKQKILFNY

>BmorOBP36

MAVSEISRILTFLTIVSFIYIVYSFKPLTKDEHIERYNKMNEDIEPFRKNLTECARQVKASMADVEKFLKRIPQSNMEGKCFVACILKRNSLIKNNKLSQENLLEVNRAVYGDDSEVMSRLKTAILECSKIVEDIFEICEYASVFNDCMHMKMEHILDKITMERRMEALGQMSSNPDEWSEEEDEMLKLVKDEL

>BmorOBP37

MFYPFRFTLLFYGLFVIYLVRAEPEKENHFTLALKKTLFSTARSCMSHVNANETDLEYLRKDPPFPDKAACIIKCLLEKIGVVKNNKYSKMGFLTAVSPLVFTNKKKLDHYKSVSENCEKEINHDQTTECELGNEVVSCIFKYAPELHFKT

>BmorOBP38

MANLVLLLTFVLMTLSMARLKSTEAPKSKTALFNDQDNMGYEELDMEEIMSACNESFRIEYAYLESLNDSGSFPDETDKTPKCYIRCVLEKTEILSENGVLNPATAALVFAGERNGKPMSDLEEMAVACADRHEKCKCEKAYNFVKCLMYMEIDKYEKKN

>BmorOBP39

MVRKISALLCCFCVLGISMCDSAISTDNEQRCKNPPTAPQKIERVITLCQDEIKLSILREALDVIKEEHTMPAERKRNKREVPFTHDEKRIAGCLLQCVYRKVKAVDGFGFPTLEGLVGLYSDGVNERGYFMAVLEASRECLMKNHDKFSRTTPMDNGRNCDVSFDIFECISDRIGEYCGTSGL

>BmorOBP40

MSEFIQPSWRTQCNFRLNWDNRNRLSIDISHGAATTQTPVPTTKPKALRDFMVVPQSCDKTTCVFKKLNIVSDKGVVDVKSFIKLLDKFTNSYPVWNSAKARVITTCLRKSLIAYDGGCELNNILACTFDVLSENCPLNGNNQTC

>BmorOBP41

MLTILFLLPIVVGVLSGNIPEQPRVYCGELPNTIYSCLGNPKIIQPEVSEKCNKPISECDKTRCIFKESGWAKNNVIDKKKVSDYFEQFAKDNPDWSAAVQNFKTTCLSDSLKPQGVDTNCPAYDIIHCALISFIKFASPSQWSTSEQCVYPRQYAGACPVCPERCFAPSVPNGSCNACLALLRTP

>BmorOBP42

MMGYACVFVILAVLQAISAEDPPGLPPFLKDAPEKCKSPPRVKNPNECCISEPFFKEADFIECGIEKPGSERGPPDCSKQNCLLKKYNLLKNDETPDIEAIKSLLDKYIEKNPSFKSSVEKAKECLREDLPGPPQICLANRMTLCIGTVLLMECPDEKWNTTDDCKAFKDHMTECQKYFPK

>BmorOBP43

MKVCVLFAIFTVAQAAKATLKPISACCNIPELGNPEPLAECSNPKLPGPCKDIQCVFEKSGFLTENKTLIKEAYKTHLRQWAKEHEGWSVAVEKAISDCVDKDLRQYLEFPCSAYDVFTCTGIAMLKKCPNEHWTC

>BmorOBP44

MSRLVLFFTILVVLQEFIINLYFNFITEIDSCCVKKYPKLFDSEFITECYNTQRKANDKCERDMCVARKLNLLTEEDSINKDALLRFVEEGFKTEIDLVNAIKKKCFEEDISNIGKPEMCEVAKYKICITSRMAEDCPKWDSKGICSSAQQKVENFMKMLS

>SlitPBP1

MANARWRFVFVVYALYLTSAVLGSQDLMVKMTKGFTRVVDDCKTELNVGDHIMQDMYNYWREDYQLINRDMGCMLLCMAKKLDLMDDQTMHHGKTEDFAKSHGADDDVAKKLVSVIHECEQQHAGIADDCMRVLEVAKCFRTKIHELKWAPSIEVIMEEVMTAV

>SlitPBP2

MAFCPSVTMSLRVALVVAASLLVVVQASQDVMKNLAVNFAKPLDDCKKEMDLPDSVTTDFYNFWKEGYELTNRQTGCAILCLSSKLEILDQELNLHHGRAQEFAMKHGADEAMAKQIVDMIHTCAQSTPDEAADPCMKALNVAKCFKLKVHELNWAPSVELIVGEVLAEV

>SlitPBP3

MGSRNVFVALVVLTVGMREIEPSKDPMKYIASGFVKVLEECKHELNMNDHLIADLFHYWKLEYTLLNRDTGCAIICMGKKLDLLDASGRMHHGNAQEFAKKHGAGDEVASQIVQIIHDCEKKHERDDDECLRVLEVAKCFRTGIHELNWQPNVEVIVSEVLTEI

>SlitGOBP1

MLLLLRALPLLAAVLPLRADVNVMKDVTLGFGQALDKCRQESQLTEEKMEEFFHFWREDFKFEHRELGCAIQCMSRHFNLLTDTSRMHHENTEQFIQSFPNGEVLARQMVELIHACEKQHDHEEDHCWRILHVAECFKQACVQRGIAPSMEIMITEFIMEAEAR

>SlitGOBP2

MTSKCCLLLVLMAAATSSVMGTAEVMSHVTAHFGKALEECREESGLSAEVLEEFQHFWREDFEVVHRELGCAIICMSNKFSLLQDDSRMHHVNMHDYVKSFPNGHVLSEKLVGLIHNCEKQFDSMTDDCERVVKVAACFKVDAKAAGIAPEVAMIEAVMEKY

>SlitOBP3

MWMQALVLTLATLATLAAAAVEMDEDMAELARMVRDNCAGETGVDVALVEKVNAGAELMPDDKLKCYIKCTMETAGMMADGEVDIEAVLALLPPSLAEHNAPAL RACGTQRGADHCDTAFRTQQCWQNANKADYFLI

>SlitOBP4

MTKVLFAIVLTMITFAVVLSASTKEAMTTTMSDQVNSIDVDVLAVMDMCNDSYRIDPTYLQALNESGSFIDETDKTPKCFIRCVFENVGIVSEDGKQFNPARAAVIFAGERNGKPMEDIADMTALCATDRQETCPCDRSYKFLRCLMSMEIERYEKS

>SlitOBP5

MSVVRCSSLLVAIFCFVSVNAISGDEEAGIKDALRPFVQECADEFGITEEQFEEAKKKASAADIDPCFMSCFLKKAEFFDSQGKFDVDSTMAFAKEHLTSEPAMKFVEAVGD ECVKINDEDVSDGDKGCDRAKLLFECIAETKKKME

>SlitOBP6

MSKFTCLVLCVVAVSLSGVHATAEEKAAFIEAVKPYVQECSKEHGVTPEDIKSAKAAGNADGINSCFLSCVYKKAEVITEKGEYDADKALEKLKKFVSNEDDYAKFANIGKKCASVNEKSVSDGEAGCERAALLTSCFLEHKSEISA

>SlitOBP7

MDQKRICLFVIAMFLASGSDAMSRQQLKNSGKMLKKNCMNKIGVTEDQIGSIDKGKFIEDRKVMCYIACIYELTNVIKNNKLNYEASIKQIDLMYPPDVKESAKAAVEKCKDVQKKYKDICEASFYAAKCMYEFKPEDFIFA

>SlitOBP8

MLLTKIVKFFILVATCEAMTMKQIKNTGKMMRKTCQP KNNAEDEKIDPISDGVFIDEKEVKCYMACIMKMANTIKNGKLNYDAAMKQADLLFPDDIKEPAKEAITACRKVADAHKDICDASFHVTKCIYNHNPGIFYFP

>HarmPBP1

MEFHRSTMMSVRLALVVAVCLFIRVDASQDVIKNLSMNFAKPLEDCKKEMDLPDSVTTDFYNFWKEGYEFTNRQTGCAILCLSSKLELLDQELKLHHGKAQEFAKKHGADDAMAKQLVDLIHGCAQSTPDVADDPCMKTLNVAKCFKAKIHELNWAPSMELVVGEVLAEV*

>HarmPBP2

MAASRWLFARAFCLVLMMGSAMSSKELLTKMTGGFTKVVDACKTELSVGDHIMQDMYNFWREEYQLVNRDLGCMIMCMTAKLDLIGDDQKMHHGKAEEFAKSHGADDALAKQLVGLIHGCETQHQAIEDHCSRALEIAKCFRTKIHELKWAPSMEVIMEEIMTAA*

>HarmPBP3

MGSRHVFFALVVLAVSVRKAEPSKDAMQYITSGFVKVLEECKHELNLNEQILADLFHFWKLEYSLLGRDTGCAIICMSKKLDLLDANGRMHHGNAAEFAKKHGAGDEVASKIVTIIHECEKKHEQDGDECLRVLEVAKCFRTGIHELNWQPKVEVIVSEVLTEI*

>HarmGOBP1

MPGVLRALLVLAAAAPLLADINVMKDVTLGFGQALDKCREESQLTEEKMEEFFHFWRDDFKFEHRELGCAIQCMSRHFNLLTDSSRMHHDNTEKFIQSFPNGEVLARQMVELIHSCEKQFDHEDDHCWRILHVAECFKGSCVQRGIAPSMELMMTEFIMEAEAR*

>HarmGOBP2

MTSKSCLLLVAMATLTASVMGTAEVMSHVTAHFGKALEE CREESGLSAEVLEEFQHFWREDFEVVHRELGCAIICMSNKFSLLQDDSRMHHVNMHDYVKSFPNGHVLSEKLVELIHNCEKKYDTMTDDCDRVVKVAACFKVDAKAAGIAPEVAMIEAVMEKY*

>HarmOBP1

MSKFTFFVLCVVAVSLSKVYASDEDKAKLHEALKPLVEECMKDHEVSLDDLKAAKEAKSADGVKPCFLACVYKKAEVLNDKGEFDADHALEKLKEFVSDEDVLAKVAEVGNTCKAVNDKAVSDGDAGCERAALLTACFLEHKAEILV*

>HarmOBP2

MMDRKRLCLLIIAMFLAQGSDAMSRQQLKNSGKMLKKNCMNKNQVTEDQIGSIDKGKFVEDKKVMCYIACIFEMTNVVKNNKLNYDASIKQIDLMYPPDLKESAKAAVEKCKDVQKKYKDICEASYWTAKCMYDFKPEDFIFA*

>HarmOBP3

MSKFTCFVLCVLAVSLGEVRSNALEKAAIRAAVYPLIVDCAKEHAVTLEQLKAAKASHSAEGINPCFQSCVYKKTGIFNDNGEYDVANAKTKLQKFVTDEDEYARIAEVGKTCASVNDKSVSDGAAGCERAALLTACFLEHRAQIII*

>HarmOBP4

MSKLTCVVFAAVAVVFSNVNADDETRASFRQVLGPLVMECRNEFGITEDDLKKAQQERSPDALKPCFIACVFKKFGIITSAGKYDSDASISRIKDVVKNDDLLAKLKSVGEKCNSVNDASVSDGDAGCERAALLAKCFIENKSELSI*

>HarmOBP5

MSKFTCLVLCVVAASLSQAYASEEEKAAFREAIKPIVEECSKEHGVSHDELKSAKDNQNADNIKPCFLGCVYKKAEVFNSKGEYDVDKALEKLKKFVSNDEAYAKFAEVGKKCASVNDKAVSDGDAGCERGALLTACFLEHKAEVPL*

>HarmOBP6

MSKFTCLLLCVVAVSLSKVHATEEEKEAIRAAVRPIMQECGKEHGVTLDDLKAAKAAHSADGIKPCFQSCVYKKAGIFNDNGEYDIANAKTKLQKFVTNDEEYARIAEVGKMCASVNDKPVTDGAAGCDRAALLTACFLEHRAQIII*

>HarmOBP7

MFRFGVLSFVVLLFCMESSYALSSEEELSIKEALHPFVVECAEEYGMTEEMFEEAKKKGSAEDIDPCFMSCFLKKTGFFDDSGKFDAEKSISFAKEHITSESAIKFLEAGAGECVKINDEDVSDGENGCDRAKLLFDCLTELKKKMSE*

>HarmOBP7.2

MSRFGVLSFVVLVFCMENIYALSSEEELSIKEALHPFVVECAEEYGMTEEMFEEAKKKGSAEDIDPCFMSCFLKKTGFFDDAGKFDAEKSISFAKEHITSETAIKFLEAGAGECVKINDEDVSDGDKGCDRAKLLFDCLTDLKKKMSE*

>HarmOBP8

MLLIEIVKFLTLVAMCEAMTMKQIRNTGKMMRKSCQPKNNVADEQIDPIAEGVFNEDKEVKCYMACIMKMANTIKNGKLNYEAAIKQADLLLPDDIKEPAKEAITACRKVADAYKDICDASFHITKCIYTQNPGIFYFP*

>HarmOBP9

MCKFSVLFLYSAVMAVNIWSASCISEEDKAAIITAIAPLAQNCGSECGLDNDDFEKYKEDGSDMDPCFKACLMTQMGVLDKEGKYDGKGLHKAMEEADYPGDKDDAQKFLDELDRCFDAKGDNSGSDEEAKMKRADVLFRCMQDMKEK*

>HarmOBP9.2

MCKCSVVFLYLAVMAINIWRASCLSEEDKAAIITAIAPLAQNCGSECGLDNDDFEKYKEDGSDMDPCFKACLMTQMGVLDKEGKYDGKGLHKAMEEADYPGDKDDAQKFLDELDRCFDAKGDNSGSDEEAKMKRADVLFQCMQDMKEN*

>HarmOBP13

MFTGTLPLVVFLATFAYGGKEKPVFSDEIKEIIQTVHDECVAKTGVAEEDITNCENGIFKEDPKLKCYMFCLMEEASLVDDDDAVDYDMLVSLIPEEYVDRTTKMIFSCKHLDTPDKDKCQRAFEVHKCSYEKDPDLYFLF*

>HarmOBP18

MKSFVVFCVLVAGAFAANVSLPPKQNEKANQIATECMKESGLKPEVLAEAKKGHISDDEHLKKFTFCFFKKAGIVSEDGKLNTEVALAKLPPGVDKAEAEKLLETCKGKTGKDVTDTVFEIFKCYHHGTKTHILLGF*

>HarmOBP15

MGSRHVFFALVVLAVSVKKEKPSKHPMPYITSRFVKVLEECQHELKLNEHILEHLFHFWKLEYSLLGKDPGCAIICMSTKLDLLDLYGRMHRGNAAEFAKKHAAGDEVPSKIVTIIHFCQKKHEQDGDECLQVLEVATCCRTGLHDLNWQHQVEVIVPDVLTEI*

>HarmOBP16

MFKLCVVLAFIVATCHGGTLERTSSTCGQIPRELTACLDLQPAVSPEIQEKCRRANECERLTCVFREYNLLDGAEVNKERTAAFLDNFVKQYPSWEVAIDVAKTSCLRSSGLKPQGVFLDCPAYDIIQCVFANLVKNALPSQWSSMSQCNHAREFAAACPICPDACFAPLVPIGTCNACSAARRSS*

>HarmOBP17

MRAWSVTLVALLGALGAARAVAMDEDMAELARMVRENCAAETGADVALVERVNAGADLMPDDKLKCYIKCTMETAGMMADGEVDIEAVLALLPPELAEHNAPSLRACGTVRGADHCDTAFRTQQCWQNANKADYFLI*

>HarmOBP18a

MTRQQLKNSGKLMKKSCMPKNDVTEEEVGDIEKGKFIESRNVMCYVACIYTMTQVVKNNKLSYEAVIKQVDMMFPAEMRDAVKAAATSCKDITKKSKDLCESAYWTAKCMYDYDAENFVFP*

>HarmOBP19

ARTEHEIKEWLFREGVACNKDFPITPDEMMMLKDNKLP DSTNAKCLIACIFKKTGMIDSKGMFDPDKSIAMTEKDFADNPEKLATSKKLMEACRGVNEQAVADGEKG

>HarmOBP20

KVFYLLTVLSACYGAVDITKYFKTCNRNAIDVNDCMADAVQKGIAVMINGIDELGIPPIDPYLQKDFRLEYKNNQIAAKLNMKNIQVEGLRAAKVHDARLRADDDKFHLEVDLTSPKVTVHAEYHGEGKFNSLRILAFGEVNTTMTDLVYTWKLDGVPEKNGTETYIRIKEFYMRPDVGSIVTNFKNDNPESRELTDLGTRFANENWRTLYREFLPYAQANWNKIGTKVANKLFLKVPYDQLFPTSS*

>HarmOBP21

FQMSRAQVKKTMSLVKNQCMPKNSVTEDQVGKIEEGVFLEDRNVMCYVACIYKNLQVVKNDKLDMSLITKQIDALYPPELKEPVKKAVSLCIHSQDNYNDLCEKVFHASKCLYEKDPASFIFP*

>HarmOBP22

MTREQIKNSGKLIKKTCMAKNDLSEDQVKDVDKGKFIEEKPFMCYIACVYKMGQTIKGNTVNHDMMIKQVEMMFPNEMKAPMKAAIEHCRPVVKKYKDVCEVSYWTAKCIYEFDPPNFMFP*

>HarmOBP31

MSKFTCIVLCVVAASLTKNTHAAISEEEKEAFRAAMAPILAECSEEHGVSEKDIEAAKESGNADDIKPCFLGCLMKKTETLDAKGLFDAEKGLSQLKKFIKDDEDLAKFEKIGNICKSVNEKAVSDGEEGCERAKLLLACFLEHKAEMPF

>SexiPBP1

MAGAKWQFVCVVFALYLTSAALGSQELMMKMTKGFTKVVDDCKAELNAGEHIMQDMYNYWREDYQLINRDLGCMILCMAKKLDLMEDQKMHHGKTEEFAKSHGADDEVAKKLVSIIHECEQQHAGIADDCMRVLEISKCFRTKIHELKWAPNMEVIMEEVMTAV*

>SexiPBP2

MAFCRSATMSVRVALVVAASMLVVVQASQDVMKNLAINFAKPLDDCKKEMDLPDSVTTDFYNFWKEGYELTNRQTGCAILCLSSKLEILDQELNLHHGRAQEFAMKHGADETMAKQIVDMIHTCAQSTPDVAADPCMKTLNVAKCFKLKIHELNWAPSMELIVGEVLAEV*

>SexiPBP3

MGSHNVFVALVLLAVGMRVAEPSKDAMKYITSGFVKVLEECKQELNMNDHIIADLFHFWKLEYALLSRDTGCVIICMSKKLDLLDANGRMHHGNAQEFAKRHGAGDDVASKIVQIIHDCEKKHERDDDECLRVLEVAKCFRTGIHDLDWQPKVEVIVSEVLTEI*

>SexiGOBP1

MLFLLRALPLLAAVLPLRADVNVMKDVTLGFGQALDKCRQESQLTEEKMEEFFHFWRDDFKFEHRELGCAIQCMSRHYNQLTDSSRMHHDNTEQFIKSFPNGEVLARQMVELIHSCEKQYDHEDDHCWRILHVADCFKQGCVQRGIAPSMEMMMTEFIMEAEAR*

>SexiGOBP2

MTAEVMSHVTAHFGKALEECREESGLSAEVLEEFQHFWREDFEVVHRELGCAIICMSNKFSLLQDDTRMHHVNMHDYVKGFPNGHVLSEKLVELIHNCEKRFDSMTDDCERVVKVAACFKVDAKAAGIAPEVAMIEAVMEKY*

>SexiOBP1

MSKFTCLVLCVVAGCLSGVHATAEEKAALIEAVKPYIQECSKEHGVTPEDIKSAKEAGNADGINACFLRCVYNKAGVINDKGEYDADKALEKLKKFVSNEDDYAKFAEIGKKCASVTETSVSDGEAGCERAALLTSCFLEHKSEVHA*

>SexiOBP2

MKSFVVFCIVLVVGVCANEKGNKLDRPFASECIKETGVKNELLEEAKKGIISEDPAFKAFTYCFFKKIGIVGEDGLLNRDVAIAKLPSGVDKSEAEKLLDSCKSKTGKDAVDTVFEIFKCYQQGTKSHIMFAS*

>SexiOBP3

MVKLTCVVFCAVAMALSVFVAGEDANSVFQGAIKPLIAECAKEYKLSDEELLKNRGLAGLSNLPPCFIGCVLKKFDIINDKGLYDAEAGIAKIEKLLPNNEFLDKISGVLKSCESANEKSVGDGDAGCERAVLVATCYLEHKTAVIA*

>SexiOBP4

MWNFLVVFLAICSCVYGLTEEELKMEFTKLIMKCNKDGKVDMTELVQLQNYVVPTKQTTKCVLACAYKAAEVMNAKGEYDIDHAYKVAEMMKNGDEKRLVNAKKMADLCVKVNEQSVSDGEKGCDRAAMIFKCTVENAPKFGFKL*

>SexiOBP5

MTMKQIRNTGKMMRKTCQPKNNVEDEKIDPIAEGVFIDEKEVKCYMACIMKMANTIKNGKLNYDAAIKQADLLLPDDIKEPAKEAITACKKVADAHKDICDASFHITKCIYNHNPGIFYSP*

>SexiOBP6

MLGSLLFVFAFSVFSLGAEALLIDDLKQKYADSILQCSQQYPLDRADAELLQNKVMPDKESTKCLFACVYKVTGVMSDQGELSVEGVNALSQKYLADDPEKLKKSEEFTEACRTVNDAPVSDGARGCDRAALIFKCTIEKSPDFSFV*

>SexiOBP7

MTKVLFAIVLMMITFAVTLSASTKEAMTTTMTDQVNSIEVDVLAVMDMCNDSYRIDPTYLQALNESGSFIDETDKTPKCFIRCVFENVGIVSEDGMQLNPARAAVIFAGERNGKPMEDIADMTALCATDRQETCPCDRSYKFLRCLMSMEIERYEKS*

>SexiABP1

MSVVRYSSFVVALFCLVSVNAMSGDEEAGVRDALRPYVQECADEYGITEEQFEEAKKKASADDIDPCFMSCFLKKAEFFDAQGKFDVDSTMAFAKEHLSSEPAMKFVEAVGDECVKINDEDVSDGDKGCDRAKLLFDCIAETKKKMD*

>SexiOBP10

MDRKRICLFVIAMFLASGSDAMSRQQLKNSGKMLKKNCM NKIGVTEDQVGSIDKGKFIEDRKVMCYIACIYELTNVIKNNKLNYEASIKQIDLMYPPDIKESAKAAVEKCKDVQKKYKDICEVSFYAAKCMYEFKPEDFIFA*

>SexiOBP8

MARRQQGAMFTETLPLFVILVAVTHGGKDKPVFSDEIKEIIQTVHDECVAKTGVAEEDITNCENGIFKEDAKLKCYMFCLLEEASLVDDDDTVDYDMLVSLIPDEYYERTTKMIFACKHLDTPDKDRCQRAFEVHKCSYEKDPDLYFLF*

>SexiOBP9

MKTLFVFAACILLAQALTDEQKEKLKKHRTECLSETKVDEQLVNKLKGGDYKTESEPLKKYALCMMMKSELMTKEGKFKKDVALAKVPNPADKPTVEKLIDACLANKGNTPHQTAWNYVKCYHEKDPKHAIFL*

>SexiOBP11

MKEGNRYSHERRITNDSGDQLMVINATDDDYSGYGSGNMGEKLLTSVPRPASSSNNINKNNTRRTRRNEPFLNRPDSDQCLSQCVFANLQVVDSRGIPREAELWNKVQTSVTSQQSRSALHDQIRACFQELQSEAEDNGCSYFNKLERCLMLRFSDRKVDGKGNPKKSSTEQT*

> SexiOBP26

MSKFTCIILCVVAASLTKVSHAVTEEEKEAFREAMAPIIAECSEEHGVSEADIKAAKEAASADGIKPCFLGCVMKKIEVLDSKGLYDAETGLGKLKKFVKDDDEFAKFEDIAKKCLKVNDESVSDGEAGCDRAKLVLGCFLEHKVEMPF

>AipsPBP1

MAPHPSVTMYVRLALVIIAGLFITVECSQEIIKNLSLQFAKPLEDCKKEMDLSDTVITDFYNFWKEGYEFTNRQFGCAILCLSSKLELLDQDLKLHHGKAQEFAKKHGADEAMAKQLVDMIHSCTQSTPDVADDPCMKTLNVAKCFVAKIHDLKWAPSMDLIMGEVLAEV*

>AipsPBP2

MAASRWCIACLVCVLFAARSVMTSQEVVASFSKGFTNVVEHCKAEVNAGEHIMQDIYNFWREEYQLVNRDLGCMVLCMANKLGLIGEDQKMHHAKAEEFAKSHGADEAVAKQLVAILYECETKHAAVEDECGMALEIAKCFRTKMHELKWAPSMEVAMEEIMTAV*

>AipsPBP3

MGTYNVFFAFVLMAAGVREIEPSKDAMKYITSGFVKVLEECKQELNMNDRIIADLFHYWKLDYTLLNRDTGCAIICMSKKLDLLDDTGRMHHGNAQEFALKHGAGEEVASKI VTIIHDCEKKFERDDDECLRVLEVAKCFRTGIHDLDWQPKVEVIVSEVFTDM*

>AipsGOBP1

MTQPGQVLVLVLLAAAALADVNVMKDVTLGFGQALDKCRQESDLTEEKMEEFFHFWRDDFKFEHRELGCAIQCMSRHFNLLTDSSRMHHVNTEEFIQSFPNGEVLARQMVALIHGCEKQFDHEDDHCWRILHVAECFKHACVAHGVAPSMEMMMTEFIMEAEAR*

>AipsGOBP2

MTLRCCLLLVVVAAVTRSVVGTAEVMSHVTAHFGKALEECRDESGLSAEVLEEFQHFWREDFEVVHRELGCAIICMSNKFSLLQDDSRMHHVNMHDYVKGFPNGEVLSGKLVELIHNCEKQYDTLTDDCDRVVKVAACFKVDAKAAGIAPEVAMIEAVMEKY*

>AipsOBP1

MDISKRRSKNAFRRLLVNTWLRLVQIFTCLSAPPVVSADVTSKCQGSKYENECDKLTCVFRKAKWLDGNAVDKAKLITYFEQFEKDHPEWAPAMQNVKTSCLGAELKTQGVFLNCPAYDVMHCVLGSFIKHATPTQWSTSASCSYPRAYAAACPICPEDCFSAQVPFGSCNACYLPPRTP*

>AipsOBP2

MSKFTCLVLCVVAASISRVHADDDANKAAFREAFKPILDECSKEHGVSNDDIDAAKKAGSADAIKPCFFGCIYKKAEVFNAKGEYDVDSALSKLKKFVPDEAKFAKYAEIGKKCASVNEKPVTDGDAGCERGAMLTACFLENRAEMLI*

>AipsOBP3

MIRSCRCLVFAAVFQVVLGQGLTGTDSGPPGFQRPQSYVPKHCFAPPPGVDLHTCCPIPQLFPDEDMESCGIQKLTKEQYENPSPARIPCQESICLLRNANLLKQNNSIDYEKMGDFVDNWAKMDPDFTIPITNAKKVCLIEGGPPAPPVCEPDRIFTCLTSYVLWNCKLRLDSGEGCKILKEHMDGCRPFLAGP*

>AipsOBP4

MFGYQFLSFAAALICFGSSYALTSEEEANIKEAFHPFIMKCAEEYGITEEQFEEAKEKHSAEGIDPCFMSCFMKESGFFDSAGKFDADKTKEFVDAHLTSERAITFMEAVGSECAKVNDEEVTDGDKGCDRAKLMWGCIQDLKEKMEGSE*

>AipsOBP5

MKYFVLFVALVAGIHANVTLPPEQSEKALKTASECIKETGVSKEVLAEAKKGHIADDEGLKKFTLCFFKKAGIVDNDGKLNLETALAKLPPGVDKAEAKKVLEGCQAKSGKTPQDTAFEIYKCYHAGAKTHIALAGI*

>AipsOBP6-PG

QRENKGASLKPLSVCCDIPELGDPKHLAKCSNPKLPGPCNDVQCVFEESGFLTDKNTLNKEAYRNHLKQWEENNKGWTVAVDKAIKECVDNDPRQHLDIPCKAYDVFTCTGIAMLKKCPDSAWKC*

>AipsOBP7

MSKFTCVLCVVALSLSSVYVTRAHKPNLRDAWRSELDECAKEYPVTNDEIDTAVRSGDSSNLNPCFNFCVFNKTGFFTENGEYDLKNGLIKLRKAIRDDEEYTKFEEVATECTEDKNTSCDEKAKCDSANRLSLCFLRFKDKVRI*

>AipsOBP8

MYLRSTNGGVRSFPLGESAYTTKIVEICSKETGLKKQVPPEEKEIKFSQRKGLREFNDCYLAKTGVTTSDGKLNIDEALEKLPPGFAKPFVEHCQANIILGYIEENVNDFSTCFHQEVQNHLLSFYGFENYWVMLVLGTSFDKTRFTTLFFDKHFDFWLAERAGFVNL*

>AipsOBP9

VFICGVLSLNVKASSLDELKMKYVEMIIECSDTYPITAADTLQLKTKTMPDNESIRCLFACVYKKAGMMNEQGELSVEGVNEMTRRYLSDDPDKIKKSEQFTEACKSVNDVPVSDGTRGCDRAALIFKCTVEKSPDFDLL*

>AipsOBP11

MTYKVFILVFLTYVSLATSALAPFITKCKWDDSKCIKESAQKVIPLFADGIPDLHVEKHDPLLIKRVDASSPNLKLIVTDIEVKGLKNCEAKKITRDLKAMKLSVKFLCAVDFKGVYDMKGQLFVLPIEGNGDLTAHVPKIQLNAEVDMVDKTGKDGKKHWGVKSWRHSFELKEKSNVKFENLFPDNEFLRKTTEELIASNGNDVIVEVGPEIIKAVTAKVIESIKKLFDEVPVEELAIDE*

>AipsOBP12

MYSGTIFLFSFILLIVSNVTFVSSQMTREQVKNSGKLVKKTCSAKNDLTEDEVKDVDKGKFIEEKKFMCYVACVYKMGQAVKGNSLNHDMMIRQVDMLFPADMKAPVKAAIE HCRPVAKKYKDICEASYWTAKCVYEFDPPNFMFP*

>AipsOBP13

MVLIYIVKFLILVAMCEAMTMKQIRNTGKMMRKSCQPKNNVEDEKIDPIAEGIFIDEPEVKCYMACIMKMANTLKNGKLNFDAALKQADLLLPDDIKEPAKEAIIACKKAAEGHKDICDVSFHVTKCIYNQNPGIFYFP*

>AipsOBP14

MFDPKTVFYLLTVFSVCFGAVDIRKYLKVCDRNAIDVSDCLTDAVQKGIAVMVNGIEELGVPPIDPYLQKEFRVEYNNNQIAVKMVIKNIYVEGLKDAKVHDARLRADDDKFHLEVDMTSPHVFVKAHYHGEGQFNSLKVVAYGDFNTTMSDLVYTWKLDGVPEKNGSETYVRIKEFYMRPDLSSIVTSFRNENPETRELTELGARFANENWRTLYKEFLPYAQANWNRIGVRIANKLFLKVPYDQLFPSSS*

>AipsOBP15

MDHNRLCLLVIAMFLATGSDAMTRQQLKNSGKILKKNCMNKHQVTEDQIGTIEKGKFVEDKKVMCYIACIYELTSVIKNNKLNYESSLRQIDIMYPADLKESAKAAVENCKDVQKKYKDICEASFHTAKCMYDFKPEDFIFA*

>AipsOBP16

MFPGSIPFISGCVHLGVSNYFRSTQSNLVVHYEDDQIVDAIYNCQDENGFDEVLSNSTNLEENFPEKEGLKKSNDCFLKKTGFVTSDGKLNIDKTLEKLPPSFVKPIVEHCQANIALNYTTESVENFSSCYHDGILNHIFAATEVGIFPFIQTWKFFVPGTSFADTILILN*

>AipsOBP17

MNQLLVFVLIVACVRISNGMTREQVKKTMTVIKKQCMPKNSVTEDQIGKIEQGVFNEDRNVMCYVACVYKSLQVVKNERLDLGLISKQIDALYPPELKEPTKKAVSQCINIQDSYNDLCEAVFHSVKCLYEKDPATFIFP*

>AipsOBP18

MKTLFVFAACILLAQALTDEQKEKLKKHRTECLTETKVEEALVNKLKGGDYKTESEPLKKYALCMMTKSELMTKDGKFKKDVALAKVPNAADKPSVEKLIDACLANKGNTPHQTAWNYVKCYHEKDPKHAIFL*

>AipsOBP19

MFTGTVPFVLCLVAVAFGGKDKPVFSEEIKEIIQTVHDECVANTGVAEEDITNCENGIFKEDPKLKCYMFCLMEEASLVDDDGTVDYDMLVSLIPDEYYERTTKMIFACKHLDTPDKDKCQRAFEVHRCSYEKDPDLYFLF*

>AipsOBP20

MLVINATDYDYEGYGTGNMGEKLLTSVPRPASSSNNINNNDTSRTRRSEPLLNKPDLDQCLSQCVFANLQVVDSRGIPREAELWNKVQSSVTSQQSRSALHDQIRACFQELQSEAEDNGCSYSNKLERCLMLRFSDRKVEGKASTPKPASTEQS*

>AipsOBP21

MLKFSVVCLYFSVAAVNFWNVHCISEDEKKAFIEAMKPMVEECGSDCGLTEEDYKKHSKGEDMDPCFKKCMMQKLGFLDEDGKYNRKQLHESISEYTGDKDEAKRVQEQLDSCFDANGDNDGDDEESQMKRVDVLFKCLKEIKE*

>AipsOBP22

MSMWFRAMVVVGALAAARCGVVMDEDMAELARMVRESCVDETGADVKLVEAVNGGADLMEDDKLKCYIKCTMETAGMMSDGEVDIEAVMALLPPEMAEHNGPALKSCGTQRGADDCDTAWKTQVCWQNANKAEYFLI*

>AipsOBP23

MSKFTYLVLCFVAVSRVYANEDERAAFHEAAKPILVECSKENGVSFDKLKAAKEAGSADGIDPCFFSCVFKKTGVFNSKGDFDLDNSLTKLKEFVSNDEDYAKVAEVGKKCE SVNEKDVSDGEAGCERASLLTACFLEHRAEIPV*

>AipsOBP24

MAKLLLAMILTVMTFALTMSATTKDAGTKEAIMTTTVANQDSSIDSNDVDVLAVMNVCNESFRIEMSYIQALNESGSFVDETDKTPKCFIRCVFENVGIVSEDGRMFNPARAAVIFAGERNGKPMDDIADMTALCAADRKETCPCDRSYQFLRCLMSMEIERYEKS*

>AipsOBP25

SRKLREAMRPIIEQCSKEHGVTDADIQASKDSNNAASLPDCFNHCLFEKSGFIDKNGRYDRDSGLKNLSKYLKDVNQYNKVVEVTKECASVEEKPATGCELGTRLTACLLDHQTSILI*

>AipsOBP26

MSKFTCIVLFVVAASLTKVTQAVSEEEKAVAREAMAPILAECSKAEGVSDEDIEEAKKNPSVDAVNSCFIRCVMRKTDALNEKGLFDSDAALAKIRPFVKSDEDFAKFEEIGKACMSVNDKEVSDGEAGCDRAKLLLACFLEHKAEMLY*

>AipsOBP27

DSAISADAESRCRNPPTAPQKIERVITLCQDEIKLSILREALDVIKEEHTMPAQRRRDKREVPFTHDEKRIAGCLLQCVYRKVKAVDGYGFPTLEGLVGLYSDGVNERGYFMAVLEASRECLMKNHDKFSRTMPMDNGRNCDVSFDIFECISDRIGEYCGTSGL*

>AipsOBP28

MTHIFSSFIPYMITVSMFSFPVSVKIISPTVPVTVVSSSVIITFMFDMYRLVILSIVAVTTVVADTDLQECRRLVHPHSMRCCKKSADAKEKMMKNDDLKECFDLPKDPVKCEHELCMAKKKGITTSDDKLDKAKFEEVVTKDIDDKDLVADIKANCINGDLTKYGPPDFCDFVKMRHCMSMQILNHCTEWNDFGDCPQLKSIIGDCVKLVAA*

>SlitOBP9

MCLVKYHVLVLCVILVGSYALNCRSSGGPKEAELKNIYKKCLKMQEGKNSSKGNSAQDWKEPRVQIQRNDWDRGRVGSKENKNSRDDSRSGSKDKKGDSGMRDNRNDMMSRRDDMMSRGDERNDNRKHRTDDRMGNDNDRSGNRGRGNKNNRNDMNGGRDDRFGRDDYFNGREDFPQSDEYGGDMGQYNNNYYSTTQSSRRYKRERRPSNSGQRSQYNPNNHKISGYEDNFRSDERNTTDNNSSKETDNKSCALHCFLENLEMTGEDGMPDRYLVTHAITKDVKNEDLRDFLQESIEECFQILDNENTEDKCEFSKNLLICLSEKGRANCDDWKDDLTF*

>SlitOBP10

MVRKISGLLCCLCVFGISFSDSAISADSESRCRNPPTAPQKIERVITLCQDEIKLSILREALDVIKEEHTMPAQRRRDKREVPFTHDEKRIAGCLLQCVYRKVKAVDGYGFPTLEGLVGLYSDGVNERGYFMAVLEASRECLMKNHDKFSRTVPMDNGRNCDISFDIFECISDRIGEYCGTSGL*

>SlitOBP11

MKSFVVFCIVFVVGVCATEKGNKIASECIKESGVKSDVLAEAKKGNLGDDPAFKEFTYCFFKKVGIVGEDGKLNRDVAIAKLPSGVDKAEAEKLLDSCKSKTGKDAVETVYEIFKCYQHGTKSHIMFAS*

>SlitOBP12

MKTLFVFAACILLAQALTDEQKEKLKKHRTECLTETKVDEELVNKLKGGDYKMDNEALKKYALCMMMKSELMTKDGKFKKDVALAKVPNPADKPTVEKLIDACLANKGNTPHQTAWNYVKCYHEKDPKHAIFL*

>SlitOBP13

MITSCLLVLSAVVQVLLAKQPVFESGPPEPWGPPERTSHPGQFQPRVPKRCWVPPQRINVYNCCPIPTLYPDEDMQSCGFEKLSENKPQKPVYRPEGTCKEGYCVMGKFDLLLANNSVDYVKFREYLDNWAESYPEFANAIHIAKEECAQDGGPEVPPICEPDKLFLCLTSTIFWNCKLRDGEGCAALQEHMNECKQYYTRVMAPTIKDFEVR*

>SlitOBP15

MYSINCFIFSVILIVMFDNCFVYSMTREQIKNSGKLIKKTCSAKNDLTEDEVKDVDKGKFIEKKDFMCYIACVYKMGQSVKGSTINHDMMLRQVDMMFPNDMKAPVKSAIEHCRPVAKNYKDLCEASYWTAKCIYDFDPANFMFP*

>SlitOBP16

MYRFVILSIVLVSALADDIDIRECGRIFHPPPHGCCKANNAVKNKDMLAEELKDCFDGSGPKDPMKCEIDLCIAKKKGFATDDGKLDIKKFEEVITKEVGSDKDLLDEIKTNCINGDLNNYGPPEFCDFMKIKHCVTLHMMNHCSEWSDDGNCKVVKELVGKCAKVI*

>SlitOBP17

MKTFRLLCCILSIFLFFDQSYGMTRQQLKNSGKLMKKSCMPKNDVTEDEVGDIEKGKFIETRNVMCYIACVYTMSQVVKNNKLSYEAVIKQVDVMFPAEMRDAVKAAATHCKETTKKYKDLCESSYWTAKCMYDYDAQNFVFP*

>SlitOBP18

MFKLCVFLALGFVACHGAPNSSPGTPNANPGTYCGVTPDNIYRCLNNPRVVTPEVSTKCGSQFTECEKMTCIFRELKWSKRGAIDKAKVRAYFDQYETEHPEWAQAVQHVKAFCLASELRAQGVFLNCPAYDIMQCVLASFIKHASPSVWSTATDCAYPKAYAADCPVCPSDCYSPQIPFGSCNACYTQPRTV*

>SlitOBP19

MFRRTLLLFSIIYISACNGQTEAPEKNRMMGIDAVHDNNVKIDKDTIITRNLKLEKRSRGPKSVSNKNEDQIEPDWSYANFPKEVSEHVEKFKKNMTECLKEVQTSDKRPVKRLSPKMESPVHGECLIACVLKRNGVIINGKVNKDNLIALVSKFYSKDTRLMKKLEKNLDRCIEMSVRAQDDCALALVLNDCTNDLMASNKHKIMVNY*

>SlitOBP20

MEKILIFTFITLSGFAHARISVMYAHDKLSDLVAQQCLSEMYPKNKRIEIQESDEPCIIFCVLKKFGIISASGVINLDIYRKRVQIAHQLDQKTSIMDYGGSCMENAEATQHKQDVCKKAKVFNDCTHLYRILLM*

>SlitOBP21

MARRQRGAMFTEALPLFVILVAVTHGGKNKPVFSDEIKEIIQTVHDECVAKTGVAEEDITNCENGIFKEDAKLKCYMFCLLEEASLVDDDDTVDYDMLVSLIPDEYYERTTKMIFACKHLDTPDKDRCQRAFEVHKCSYEKDPDLYFLF*

>SlitOBP22

MSKFTCIILCVVAASLTKVSHAAVTEEEKEAFREAMAPIIAECSEEHGVSEADIKAAKESASADNIKPCFLGCVMKKIEVLDAKGLYDAETGLGKLRKFVKDDDEFAKFEDIAKKCLKVNDESVSDGEAGCDRAKLVLGCFIEHKVEMPF*

>SlitOBP23

MAKFSCLVLCVVAASLGSIHVASGESLRESLRPVIVACSQEHGVTDAEIQAAKDAGSPASIKPCFIACVFKKAGFINEQGQLDLETGLKNLRQFVKDDEQYKKLEEVAKKCSQVKDKAVSDGAAGCERGVLLAGCFLEHKTSIII*

>SlitOBP25

MAKVTCIVLFVVGVSLSSIQADDGKNESEVEIDVNQIIDDCIEEYHIPRRLFLAAAETGSTHALTPCFWSCCFKGVGVLNSEGQYDIDATLDLSKKIFTDHEYEKVEIIVKKCESVNGAPVSNGNIECEKSVLLADCLFDNAKKHFPNMFGVDY*

>SlitOBP27

MYKFVILCSIFVAASNADVAQTLTKRETKASLKPLSVCCDIPELADEFQLAKCSPRPPGPCEDVQCIFEVSGFLTDRNTLNKAAYRSHLQKWEKNHPGWTDSIYKAITDCVDNDPRQHLEVPCKAYDVFTCTGIAMLKKCPDTAWKC*

>SlitOBP28

MIVRFLLCLYIVEFYGAHARTDQEIKAWFFREGMDCNIEHPISPKEMLELKENKIPDTNNAKCFVACVFKKTGMLDSKGMFDAENSIAMTQKDFANDPNRLESSKKLLEACKKVNDEAVSDGEKGCERSVLLHKCFVETAPQLGIKLP*

>SlitOBP29

MWNLLVVFLAICSCVYARRRSSGAEINGLTEEELKMEFTKLIMKCNKDGEVDMTELVQLQNYVVPTKQSTKCVLACAYKAAEVMNAKGEYDIDHAYKVAEMMKNGDEKRLVNAKKMADLCVKVNELSVSDGEKGCDRAAMIFKCTVENAPKFGFKL*

>SlitOBP33

MTCSQALALLALVAISQQATTGCKNCIMLGKEEKAMFRAHSDACVAASRVEPRLVDAMLAGELLDEPALRKHVYCVLLKCKLISKDGKLQKAAVLGKMAARPDAKNATKVLESCADQTGDTPEDLAWNLFRCGYDKKALLFDYMPTNVASETDNNS*
